# Supplementary material for: Multi-sample and multi-group spatial colocalization analysis using PANORAMIC
Source: Bioinformatics. 2026 Jul 31;42(8):btag546. doi: 10.1093/bioinformatics/btag546 (PMC13472731; doi:10.1093/bioinformatics/btag546)
Supplement: Supplementary file 1 [file btag546_supplementary_data.pdf]

# PANORAMIC Supplement

July 20, 2026

## Contents

**Supplementary Methods** **3**

**S1 Edge-corrected neighborhood enrichment colocalization statistic** **3**

**S2 Spatial bootstrap implementation** **3**

**S3 Multilevel meta-analysis implementation** **4**

**S4 Simulation design** **5**
S4.1 Intrasample variance calibration simulation . . . . . 5
S4.2 Intersample degradation simulation . . . . . 6

**S5 CRC TMA preprocessing and analysis settings** **6**
S5.1 CRC data source and preprocessing . . . . . 6
S5.2 One-core-per-patient subsampling . . . . . 7
S5.3 PANORAMIC CRC analysis settings . . . . . 7
S5.4 CRC differential colocalization network analysis . . . . . 7
S5.5 CRC spatial compactness analysis . . . . . 7

**S6 HNSCC TMA preprocessing and analysis settings** **8**
S6.1 HNSCC data source and comparison definition . . . . . 8
S6.2 PANORAMIC HNSCC analysis settings . . . . . 8
S6.3 HNSCC benchmark settings . . . . . 9

**Supplementary Results** **10**

**S7 Simulation supplementary results** **10**
S7.1 Progressive degradation and breakpoint results . . . . . 10

**S8 CRC supplementary results** **11**
S8.1 CRC TMA overview . . . . . 11
S8.2 CRC cell-type composition . . . . . 12
S8.3 Full PANORAMIC CRC results at 25 $\mu$m . . . . . 13
S8.4 CRC runtime . . . . . 18
S8.5 CRC radius stability . . . . . 19
S8.6 CRC spicyR benchmark . . . . . 19
S8.7 CRC Welch $t$-test benchmark . . . . . 24
S8.8 CRC $p$-value distribution comparison . . . . . 30
S8.9 Alternative spatial statistic: cross-$L$ . . . . . 31
S8.10Sensitivity analysis . . . . . 38

**S9 HNSCC supplementary results** **38**
S9.1 HNSCC cohort overview . . . 38
S9.2 Full PANORAMIC HNSCC results at 25 $\mu$m . . . 39
S9.3 HNSCC spicyR benchmark . . . 47
S9.4 HNSCC $p$-value distribution comparison . . . 55

# Supplementary Methods

## S1 Edge-corrected neighborhood enrichment colocalization statistic

The default colocalization statistic for PANORAMIC is the edge-corrected neighborhood enrichment statistic, as described in the main text, using the generic tissue window $W$. Here, we define $W_k$ as the observed tissue window of sample $k$, with area $|W_k|$, and describe the implementation details used for self-pairs and boundary correction.

For an ordered pair $a \to b$, PANORAMIC uses the same direction convention as in the main text: $a \to b$ denotes enrichment of type-$a$ cells around type-$b$ anchor cells. For self-pairs (i.e. $a = b$), the anchor cell itself is excluded from both the observed neighborhood count and the expected target-cell count. We denote the corresponding leave-one-anchor density by $\hat{\lambda}_{k,a}^{(-i)}$, where the superscript $(-i)$ indicates that anchor cell $i$ is omitted when $a = b$:

$$\hat{\lambda}_{k,a}^{(-i)} = \frac{N_{k,a} - \mathbf{1}\{a = b\}}{|W_k|},$$

where $N_{k,a}$ is the number of cells of type $a$ in sample $k$. For non-self pairs (i.e. $a \neq b$), the anchor cell is not a type-$a$ target cell, so this reduces to the usual sample-wide density $N_{k,a}/|W_k|$. For self-pairs (i.e. $a = b$), this reduces to $(N_{k,a} - 1)/|W_k|$, preventing the anchor cell from contributing to its own expected target-cell density.

For anchor cell $i$, the expected count is

$$E_{i,a}(r) = \hat{\lambda}_{k,a}^{(-i)} A_i(r),$$

where

$$A_i(r) = |B(x_i, r) \cap W_k|.$$

Thus, the expected number of target cells is computed using the available in-tissue area $A_i(r)$, rather than the full disc area $\pi r^2$. This gives anchors near tissue boundaries smaller expected target-cell counts.

Equivalently, the anchor-level statistic from the main text can be written as

$$S_{i,a\to b}(r) = 100\left[\hat{p}_{i,a}^{\text{obs}}(r) - \hat{p}_{i,a}^{\text{edge}}(r)\right], \qquad \hat{p}_{i,a}^{\text{edge}}(r) = \frac{E_{i,a}(r)}{n_{i,\cdot}(r)}.$$

Here, $\hat{p}_{i,a}^{\text{edge}}(r)$ is an edge-corrected expected target-cell count divided by $n_{i,\cdot}(r)$.

The edge-corrected neighborhood enrichment statistic is conceptually related to graph-based neighborhood enrichment and co-occurrence analyses used in spatial omics software, including Squidpy and Giotto Palla et al. (2022); Dries et al. (2021). PANORAMIC differs in three main ways: neighborhoods are defined by metric balls at fixed physical radii; expected target-cell counts are adjusted for available tissue area around each anchor; and sample-level estimates are paired with spatial-bootstrap variance estimates for multilevel random-effects meta-analysis. The boundary correction also draws on the broader spatial point-pattern literature, where edge correction is needed because neighborhoods near the observation-window boundary are only partially observed Ripley (1976); Baddeley et al. (2015).

## S2 Spatial bootstrap implementation

For each feature $f$, sample $k$, and radius $r$, let $S_{i,f,k}(r)$ denote the anchor-level neighborhood enrichment score for valid anchor $i$. In the analyses in this manuscript, a feature $f$ corresponds to an ordered cell-type pair $a \to b$ evaluated at a specified radius.

PANORAMIC overlays the sample window $W_k$ with a regular grid of square tiles. Let $\mathcal{G}_k$ denote the set of tiles with positive overlap with the observed tissue window. Each retained tile receives the overlap weight

$$w_{g,k} = \frac{|\text{tile}_g \cap W_k|}{|\text{tile}_g|}, \qquad g \in \mathcal{G}_k.$$

Tiles with zero overlap with $W_k$ are excluded from the bootstrap sampling frame.

Each valid anchor is assigned to one retained tile according to its spatial coordinate. Let $\mathcal{A}_{g,f,k}$ denote the set of valid anchors for feature $f$ assigned to tile $g$. For each retained tile, PANORAMIC computes a tile-level summary

$$\bar{S}_{g,f,k}(r) = \frac{1}{\max\{|\mathcal{A}_{g,f,k}|, 1\}} \sum_{i \in \mathcal{A}_{g,f,k}} S_{i,f,k}(r),$$

with the convention that tiles containing no valid anchors for feature $f$ contribute a zero tile-level sum. This convention keeps the bootstrap sampling frame tied to the observed tissue window rather than redefining the set of sampled tiles separately for each feature.

Bootstrap resampling probabilities are proportional to the overlap weights,

$$\pi_{g,k} = \frac{w_{g,k}}{\sum_{h \in \mathcal{G}_k} w_{h,k}}.$$

For bootstrap replicate $b$, PANORAMIC samples tiles

$$g_1^{*(b)}, \ldots, g_{|\mathcal{G}_k|}^{*(b)}$$

with replacement from $\mathcal{G}_k$ using probabilities $\pi_{g,k}$. The replicate statistic is

$$S_{f,k}^{*(b)}(r) = \frac{1}{|\mathcal{G}_k|} \sum_{m=1}^{|\mathcal{G}_k|} \bar{S}_{g_m^{*(b)},f,k}(r).$$

Thus, overlap weights affect how often each tile is sampled.

The bootstrap-centered sample-level estimate is the mean of the replicate statistics,

$$\hat{\mu}_{f,k}(r) = \frac{1}{B} \sum_{b=1}^{B} S_{f,k}^{*(b)}(r),$$

and the within-sample variance estimate is

$$\hat{\sigma}_{f,k}^2(r) = \frac{1}{B} \sum_{b=1}^{B} \left\{ S_{f,k}^{*(b)}(r) - \hat{\mu}_{f,k}(r) \right\}^2.$$

These define the sample-level effect estimate and sampling variance used in the downstream meta-analysis:

$$y_{f,k}(r) = \hat{\mu}_{f,k}(r), \qquad v_{f,k}(r) = \hat{\sigma}_{f,k}^2(r).$$

This procedure is a PANORAMIC-specific adaptation of Loh-style spatial resampling Loh (2008, 2010). Rather than resampling individual cells, which would ignore local spatial dependence, the method resamples local spatial regions represented by tiles.

# S3 Multilevel meta-analysis implementation

PANORAMIC fits each feature independently using `metafor::rma.mv`. The input for feature $f$ consists of sample-level estimates $y_{fk}$ and corresponding sampling variances $v_{fk}$ obtained from the spatial bootstrap. The estimated variances $v_{fk}$ are passed to the meta-analytic model as known sampling variances, while random-effect terms model residual heterogeneity not explained by the within-sample bootstrap variance.

When a grouping variable is supplied, PANORAMIC estimates group-specific pooled means using fixed-effect moderators with no intercept. In matrix notation, the patient-level model can be written as

$$y_{fk} = X_k \beta_f + u_{f,m(k)} + \varepsilon_{fk},$$

where $X_k$ encodes group membership or other sample-level moderators for sample $k$, $m(k)$ denotes the patient from which sample $k$ was obtained,

$$u_{f,m(k)} \sim \mathcal{N}(0, \tau_f^2)$$

is a patient-level random effect, and

$$\varepsilon_{fk} \sim \mathcal{N}(0, v_{fk})$$

is the known sampling-error term from the spatial bootstrap.

For analyses with repeated regions or cores from the same patient, PANORAMIC can fit a nested patient-plus-sample random-effects structure,

$$y_{fk} = X_k\beta_f + u_{f,m(k)} + u_{f,s(k)} + \varepsilon_{fk},$$

where $s(k)$ denotes the sample or tissue region corresponding to observation $k$ and

$$u_{f,s(k)} \sim \mathcal{N}(0, \sigma_{fs}^2)$$

is the sample-level random effect within a patient. In the package implementation, these two structures correspond to `tau_structure = "patient"` and `tau_structure = "patient_sample"`, respectively. The nested model is implemented as a random intercept for samples nested within patients.

For two-group comparisons, PANORAMIC reports the contrast between the two estimated group means. In the package output, `beta_diff` is computed as the second group minus the first group, where group order is determined by the factor levels of the grouping variable. The standard error of the contrast is computed from the fitted coefficient covariance matrix, and the Wald statistic is

$$Z_f^{\text{diff}} = \frac{\hat{\beta}_f^{\text{diff}}}{\text{SE}(\hat{\beta}_f^{\text{diff}})},$$

where $\hat{\beta}_f^{\text{diff}}$ is a point estimator of $\beta_f^{\text{diff}}$ and $\text{SE}(\hat{\beta}_f^{\text{diff}})$ is the estimated standard error of $\hat{\beta}_f^{\text{diff}}$. Two-sided $p$-values are computed using the standard normal reference distribution, and Benjamini–Hochberg correction is applied across tested features.

PANORAMIC also supports variance flooring for non-positive or numerically unstable within-sample variance estimates. With `vi_floor = "median"`, non-positive $v_{fk}$ values are replaced by the median positive variance for that feature. With `vi_floor = "group_median"`, the replacement is computed within each group. With `vi_floor = "none"`, sample-feature combinations with non-positive variances are dropped before model fitting. Variance flooring prevents samples with zero or near-zero bootstrap variance from receiving infinite or numerically dominant inverse-variance weight in the meta-analysis.

# S4 Simulation design

## S4.1 Intrasample variance calibration simulation

To evaluate whether PANORAMIC's spatial bootstrap provides informative within-sample variance estimates, we simulated marked spatial point patterns under three regimes: uniform random mixing, opposing gradients, and clustered organization. Each simulated sample occupied a $600\,\mu\text{m} \times 600\,\mu\text{m}$ square window and contained five cell types: A, B, C, Rare 1, and Rare 2. Baseline cell-type intensities were scaled so that the expected total number of simulated cells matched the average number of cells in the CRC TMA cores.

The "uniform regime" generated each cell type from an independent homogeneous Poisson process. The "opposing-gradient" regime generated types A and B from opposing inhomogeneous Poisson processes, producing large-scale compositional heterogeneity without direct local attraction. The "clustered regime" generated $B \to A$ spatial structure using a parent-offspring process, with A cells corresponding to parent points and B cells generated around parent locations; the remaining cell types were generated from independent homogeneous Poisson processes.

For each regime, we generated 300 simulated samples. PANORAMIC was applied to each sample using edge-corrected neighborhood enrichment at radius $r = 25\,\mu\text{m}$, block bootstrap uncertainty estimation with

$B = 100$ replicates, and square tiles of side length $62.5\,\mu$m. For each feature, empirical variance was estimated across independent simulation replicates and compared with the mean PANORAMIC bootstrap variance. We summarized variance calibration using Pearson correlations.

### S4.2 Intersample degradation simulation

To evaluate PANORAMIC under degraded multi-sample sampling, we simulated nested patient-level datasets in which each observed sample was an $B \to A$ clustered point pattern in a $600\,\mu\text{m} \times 600\,\mu\text{m}$ square window with target total cell count approximately 1900. Patient-level latent effects modified the parent-process intensity, offspring abundance, and cluster spread, inducing between-patient heterogeneity in colocalization. Additional sample-level log-normal perturbations induced within-patient variability across repeated samples. Clean simulated samples were also subjected to global thinning to mimic incomplete or variable tissue sampling, while enforcing minimum total-cell and per-type cell-count thresholds.

We evaluated $n_{\text{patients}} \in \{10, 20, 30, 40, 50\}$, with 5 simulation replicates per patient-count setting. The number of samples per patient was drawn from a negative-binomial distribution with mean 2.2 and constrained to lie between 1 and 4. For each patient, 100 independent clean truth realizations were generated without bootstrap resampling to approximate the patient-specific expected colocalization effect and within-patient variance structure. Observed samples were then analyzed with PANORAMIC using edge-corrected neighborhood enrichment at $r = 25\,\mu$m, block bootstrap uncertainty estimation with $B = 100$ replicates, and baseline tile size $62.5\,\mu$m. Sample-level effects and variances were passed to REML-based multilevel meta-analysis with patient- and sample-level random effects, and estimates were compared with the corresponding simulation truth.

To assess robustness to controlled departures from clean sampling, we introduced a degradation ladder ranging from a clean baseline to mild, moderate, severe, and extreme degradation. In each scenario, contamination was assigned at the patient level and inherited by that patient's observed samples. Degradation combined quality-dependent perturbations in four components: increased sample-level heterogeneity, asymmetric thinning by cell type, directional compositional bias favoring one cell type, and inflation of the effective bootstrap tile scale. Minimum total-cell and per-type cell-count thresholds were enforced after degradation.

For each scenario and patient-count regime, we summarized PANORAMIC performance using the root mean squared error (RMSE) of the estimated pooled mean effect and patient- level heterogeneity relative to the simulated truth. Confidence intervals for RMSE were computed across the 5 independent simulation replicates. Simulation outputs also included feature-level estimates, replicate-level performance summaries, and breakpoint-style diagnostics describing how estimation accuracy changed with increasing degradation severity.

## S5 CRC TMA preprocessing and analysis settings

### S5.1 CRC data source and preprocessing

The CRC TMA dataset was originally generated by Schürch et al. using CODEX multiplexed immunofluorescence imaging of formalin-fixed paraffin-embedded colorectal cancer tissue microarrays sampled from the invasive tumor front Schürch et al. (2020). The released single-cell table includes spatial coordinates, cell-type annotations, cellular neighborhood assignments, marker measurements, and patient-level metadata. The original study profiled 140 tissue regions from 35 advanced-stage CRC patients using 56 protein markers and identified nine conserved cellular neighborhoods in the immune tumor microenvironment.

Patients were annotated according to immune infiltration patterns at the invasive tumor front. Patients with lymphoid aggregate architecture consistent with Crohn's-like reaction were annotated as CLR, whereas patients with diffuse inflammatory infiltration were annotated as DII. For CLR patients, cores included samples with lymphoid aggregate samples with diffuse regions; for DII patients, only cores with diffuse infiltration regions are sampled.

Starting from the released single-cell table, we retained cells with defined cell-type annotations and spatial coordinates. Spatial coordinates were converted from pixels to microns using the dataset-specific conversion factor $0.37742\,\mu$m per pixel. We removed undefined cells and excluded cell types that were absent from more

than 20% of images, mirroring the preprocessing strategy used for this dataset in the spicyR study Canete et al. (2022). After filtering, the processed table contained 228,811 cells across 35 patients, 140 spots, and 12 retained cell types.

## S5.2 One-core-per-patient subsampling

For the primary CRC analysis, we restricted the dataset to one core per patient to preserve patient-level biological replication. We first retained cores whose annotations matched the group-specific comparison of interest: lymphoid aggregate cores for CLR patients and diffuse infiltration cores for DII patients. From this filtered set, one core was randomly selected per patient using a fixed random seed.

## S5.3 PANORAMIC CRC analysis settings

The primary PANORAMIC run used the edge-corrected neighborhood enrichment statistic at $r = 25\,\mu$m, $B = 100$ spatial bootstrap replicates, and square bootstrap tiles with side length $62.5\,\mu$m, corresponding to $2.5 \times r$. The analysis used the block bootstrap and a convex tissue window. Features were required to have at least five relevant cells per sample; otherwise, that sample-feature combination was marked as missing. Feature-wise group comparisons between CLR and DII were fit using multilevel random-effects meta-analysis with patient-level random effects, group-specific fixed-effect means, group-median variance flooring, and REML estimation. Multiple testing correction was performed using the Benjamini–Hochberg procedure across tested features.

## S5.4 CRC differential colocalization network analysis

To summarize coordinated spatial changes across multiple cell types, we constructed a differential colocalization network from the PANORAMIC CRC differential testing results. The network was based on the primary one-core-per-patient CRC analysis using edge-corrected neighborhood enrichment at $r = 25\,\mu$m, $B = 100$ bootstrap replicates, and multilevel meta-analysis.

Each node in the network represents a retained cell type. Candidate edges correspond to ordered cell-type pairs tested by PANORAMIC. For the main CRC network visualization, we focused on CLR-enriched associations. Because the differential effect in the primary output was defined as DII minus CLR, CLR-enriched associations corresponded to negative differential effects and negative Wald $Z$-statistics. We therefore constructed the displayed network from the CLR-up component using significant features with Benjamini–Hochberg adjusted $p < 0.05$ and $Z < 0$.

Although PANORAMIC tests ordered cell-type pairs, the network was rendered as an undirected graph for visualization of higher-order cell-type organization. Edge weights were derived from the magnitude of the Wald $Z$-statistics from the differential colocalization tests. Leiden clustering was then applied to the resulting graph to identify groups of cell types participating in coordinated differential colocalization patterns Traag et al. (2019). In the plotted network, we used a Leiden resolution parameter of 1.2. Non-significant edges were optionally retained at low visual weight to preserve network context, but interpretation focused on the FDR-significant CLR-enriched edges.

The CLR-enriched network highlighted an immune/stromal module involving B cells, CD8+ T cells, CD4+ CD45RO+ T cells, and stroma. We used this cell-type set for the compactness analysis described below, which provides a sample-level spatial summary of whether the network-highlighted cell types were more tightly co-organized in CLR than in DII samples.

## S5.5 CRC spatial compactness analysis

Let $\mathcal{T}$ denote the set of cell types selected from the differential colocalization network. In the CRC analysis, we used

$$\mathcal{T} = \{\text{B cells},\ \text{CD8+ T cells},\ \text{CD4+ CD45RO+ T cells},\ \text{stroma}\}.$$

For sample $k$, let $\mathcal{I}_{k,\mathcal{T}}$ denote the set of cells whose labels belong to $\mathcal{T}$. For each source cell $i \in \mathcal{I}_{k,\mathcal{T}}$ and each target type $t \in \mathcal{T}$, define

$$d_{ki}(t) = \min_{j:c_{kj}=t,\ j\neq i \text{ if } c_{ki}=t} \|x_{ki} - x_{kj}\|,$$

where $x_{ki}$ is the coordinate of cell $i \in \mathcal{I}_{k,\mathcal{T}}$ and $\|x_{ki} - x_{kj}\|$ reprensents the distance between cell $i$ and cell $j$. When no target cell of type $t$ was available, the distance was set to infinity. Distances were capped at $r_0 = 25\,\mu$m:

$$\tilde{d}_{ki}(t) = \min\{d_{ki}(t), r_0\}.$$

The within-set spacing for source cell $i$ was defined as the average capped nearest-neighbor distance to the selected target types,

$$D_{ki}^{\mathcal{T}} = \frac{1}{|\mathcal{T}|}\sum_{i\in\mathcal{T}} \tilde{d}_{ki}(t).$$

To account for differences in overall cell density, we computed a local baseline distance

$$d_{ki}^{\text{all}} = \min_{j\neq i} \|x_{ki} - x_{kj}\|,$$

the nearest-neighbor distance from cell $i$ to any other cell in the sample. The relative compactness value for cell $i$ was

$$R_{ki} = \frac{D_{ki}^{\mathcal{T}}}{\max(d_{ki}^{\text{all}}, \epsilon)},$$

where $\epsilon = 10^{-8}$ was used for numerical stability. The sample-level relative compactness score was then computed as

$$C_k = \frac{1}{|\mathcal{I}_{k,\mathcal{T}}|}\sum_{i\in\mathcal{I}_{k,\mathcal{T}}} R_{ki}.$$

Smaller values of $C_k$ indicate that the selected cell types are closer to one another relative to the local nearest-neighbor spacing of the sample.

For each ordered pair of selected cell types, we also recorded sample-level mean, median, and standard deviation of capped nearest-neighbor distances. Pairwise distances and spatial compactness summaries were compared between CLR and DII samples using Welch $t$-tests. The primary spatial compactness comparison used $C_k$, the mean relative compactness score, and tested the one-sided alternative that CLR samples have lower relative compactness than DII samples.

# S6 HNSCC TMA preprocessing and analysis settings

## S6.1 HNSCC data source and comparison definition

The HNSCC tissue microarray cohort was obtained from Haist et al., who investigated how lymph node colonization remodels primary tumors and tumor-draining lymph nodes in head and neck squamous cell carcinoma Haist et al. (2026). The dataset includes spatially resolved profiling of primary tumors and lymph node tissues, including tumor-involved, tumor-adjacent, and benign lymph node regions. Haist et al. reported that nodal colonization was associated with interferon-$\gamma$ signaling, enrichment of immunosuppressive myeloid cells and cancer-associated fibroblasts, and spatially organized myeloid–fibroblast niches associated with T cell dysfunction and Treg activation Haist et al. (2026).

For PANORAMIC analysis, we focused on a lymph node comparison designed to evaluate spatial remodeling associated with metastatic involvement. We compared benign lymph node samples from node-negative patients with tumor-adjacent (i.e., cancer-free) lymph node regions from patients with lymph node metastasis. This setting provides a nested design in which multiple tissue regions may be observed for the same patient, allowing evaluation of PANORAMIC's multilevel modeling framework beyond the one-core-per-patient CRC analysis.

## S6.2 PANORAMIC HNSCC analysis settings

The HNSCC analysis used the same primary local spatial statistic and bootstrap settings as the CRC analysis: edge-corrected neighborhood enrichment at radius $r = 25\,\mu$m, $B = 100$ spatial bootstrap replicates, square bootstrap tiles of side length $62.5\,\mu$m, and a minimum of five relevant cells per sample-feature combination. Feature-wise group comparisons were fit using multilevel random-effects meta-analysis with patient-level or

patient-plus-sample random effects, selected according to the repeated-sampling structure available for each feature. Multiple testing correction was performed using the Benjamini–Hochberg procedure across tested ordered cell-type pairs.

We included this analysis primarily to evaluate PANORAMIC in a second real multiplexed imaging dataset with multiple regions per patient. Therefore, the HNSCC analysis is presented as a nested-design demonstration and sensitivity application rather than as a comprehensive biological analysis of metastatic lymph node remodeling.

### S6.3 HNSCC benchmark settings

To compare PANORAMIC with an existing image-level differential localization workflow, we also analyzed the HNSCC comparison using spicyR. The spicyR benchmark used the same HNSCC comparison, retained cell types, and sample inclusion criteria where possible. Because PANORAMIC and spicyR use different colocalization metrics and modeling assumptions, this benchmark should be interpreted as a comparison of differential spatial-analysis workflows rather than as a feature-identical test of the same estimand.

P-value distributions from the HNSCC analyses were compared to evaluate whether the workflows produced broadly different empirical significance patterns in this second dataset. These comparisons are descriptive and are intended to complement the CRC benchmark, where the primary biological signal and label-permutation analyses are reported.

# Supplementary Results

# S7 Simulation supplementary results

## S7.1 Progressive degradation and breakpoint results

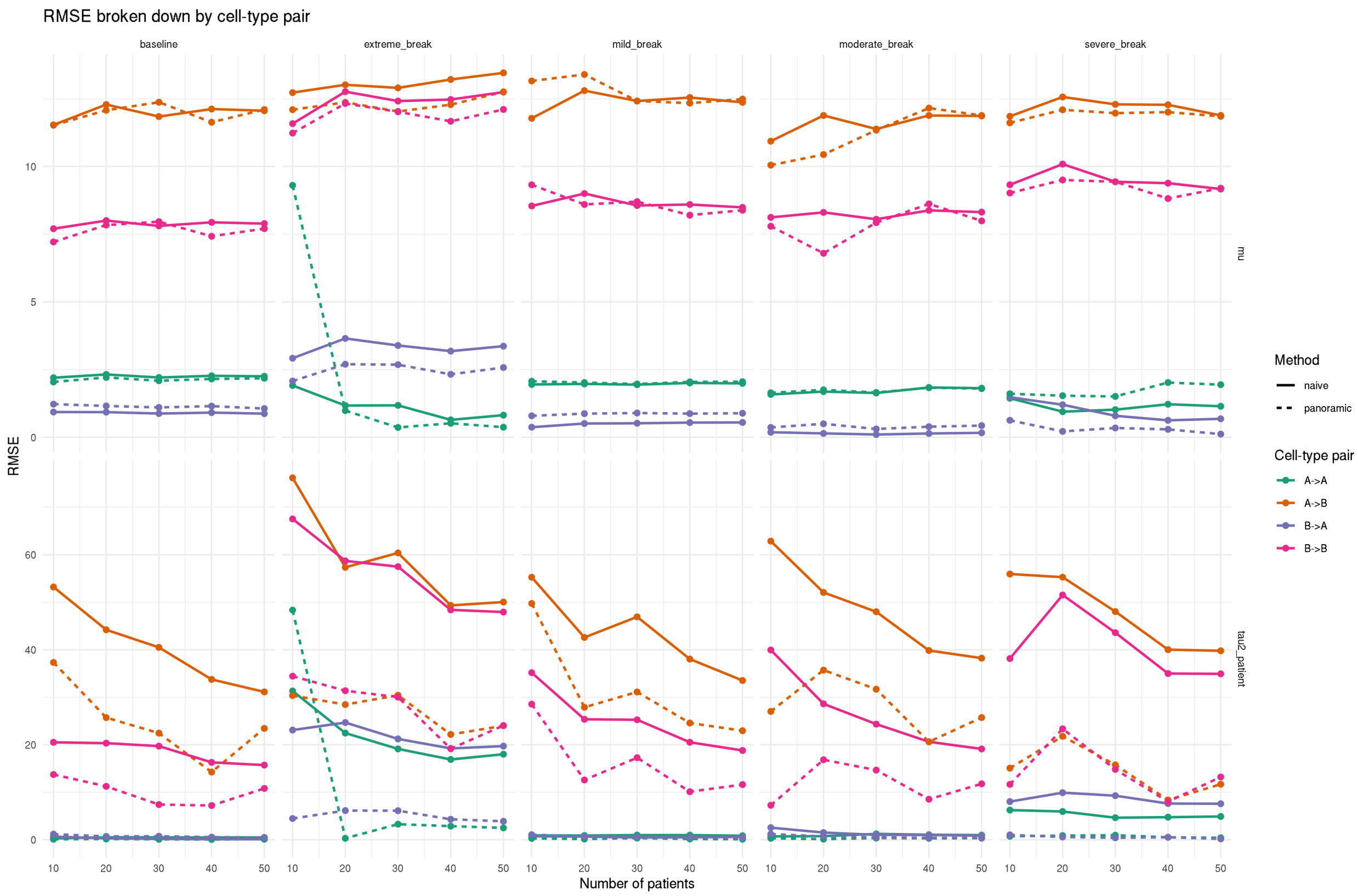


Figure S1: Breakpoint simulation performance by cell-type pair. Root mean squared error (RMSE) is shown for recovery of the pooled mean colocalization effect $\mu$ and patient-level heterogeneity $\tau^2_{\text{patient}}$ across patient-count regimes and degradation scenarios. Columns correspond to the baseline simulation and progressively degraded settings, from mild through extreme breakpoint contamination. Colors denote ordered cell-type pairs, and line type denotes the estimator: naive estimation versus PANORAMIC. PANORAMIC provides the clearest improvement for estimating patient-level heterogeneity, particularly for A→B and B→B relationships under degraded sampling, whereas improvements for the pooled mean $\mu$ are smaller and more cell-type-pair dependent. These results support the main simulation finding that PANORAMIC's primary advantage is separating within-sample uncertainty from between-patient heterogeneity under sample degradation.

# S8 CRC supplementary results

## S8.1 CRC TMA overview

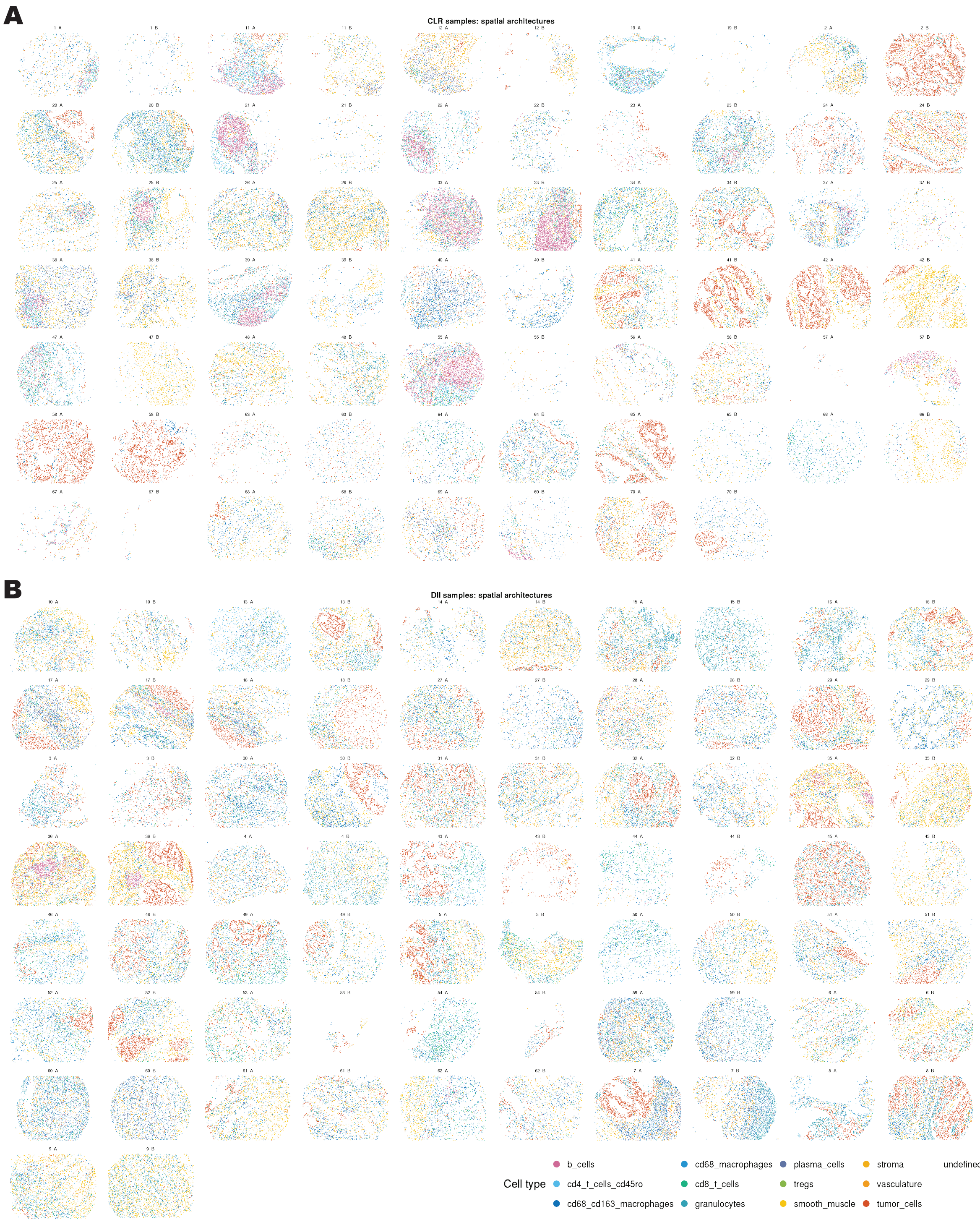


Figure S2: Visual overview of the CRC TMA from Schürch et al.Schürch et al. (2020). (A) 68 cores from patients identified to have Crohn's-like reaction (CLR). (B) 72 cores from patients identified to have diffuse inflammatory infiltration (DII).

## S8.2 CRC cell-type composition

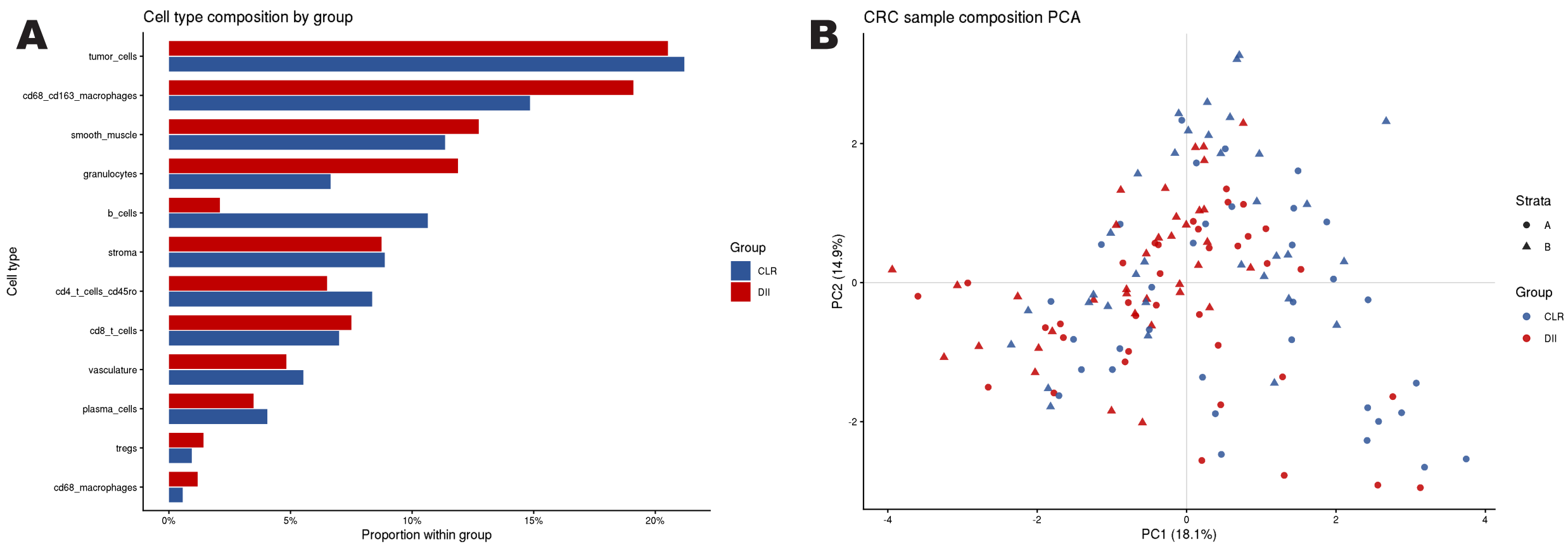


Figure S3: Overview of CRC TMA cell-type proportions. (A) Proportions of cell types between cores from CLR and DII patients. (B) PC1 and PC2 generated from the matrix of cell-type proportions per core.

## S8.3 Full PANORAMIC CRC results at $25\,\mu$m

Table S1: CRC TMA PANORAMIC results, using the neighborhood enrichment statistic at 25 micron

| Cell type pair | CLR | DII | Effect size | p-value | adjusted p-value |
|---|---:|---:|---:|---:|---:|
| b_cells → b_cells | 4.4233 | 0.1188 | -4.3045 | 0.000 | 0.000 |
| b_cells → cd8_t_cells | 2.9100 | 0.4878 | -2.4222 | 0.000 | 0.000 |
| b_cells → cd68_cd163_macrophages | -1.4564 | 0.3552 | 1.8116 | 0.000 | 0.001 |
| b_cells → cd4_t_cells_cd45ro | 2.7394 | 0.4250 | -2.3144 | 0.000 | 0.003 |
| cd68_cd163_macrophages → tregs | 0.3839 | 2.6988 | 2.3149 | 0.000 | 0.007 |
| cd68_cd163_macrophages → cd8_t_cells | 3.2809 | 7.1142 | 3.8333 | 0.001 | 0.023 |
| tumor_cells → granulocytes | 0.3400 | 1.5791 | 1.2391 | 0.002 | 0.032 |
| cd68_cd163_macrophages → cd68_cd163_macrophages | 4.0916 | 8.2103 | 4.1186 | 0.002 | 0.033 |
| tregs → cd68_cd163_macrophages | 0.0417 | 0.6038 | 0.5621 | 0.002 | 0.034 |
| tumor_cells → tumor_cells | 3.1126 | 10.3090 | 7.1965 | 0.003 | 0.034 |
| cd8_t_cells → cd68_cd163_macrophages | 1.0087 | 3.8804 | 2.8717 | 0.003 | 0.034 |
| cd8_t_cells → plasma_cells | 0.2082 | 1.1616 | 0.9534 | 0.004 | 0.044 |
| cd68_cd163_macrophages → cd4_t_cells_cd45ro | 2.6920 | 5.3203 | 2.6283 | 0.004 | 0.044 |
| b_cells → tregs | 1.2352 | 0.2767 | -0.9585 | 0.006 | 0.063 |
| cd4_t_cells_cd45ro → b_cells | 2.7578 | 1.1574 | -1.6004 | 0.007 | 0.063 |
| cd8_t_cells → smooth_muscle | 0.3121 | 1.4237 | 1.1117 | 0.007 | 0.067 |
| granulocytes → tumor_cells | 0.6000 | 1.7523 | 1.1523 | 0.012 | 0.104 |
| stroma → plasma_cells | 0.3881 | 1.5108 | 1.1227 | 0.015 | 0.123 |
| granulocytes → cd4_t_cells_cd45ro | 0.4458 | 1.4201 | 0.9742 | 0.018 | 0.135 |
| granulocytes → granulocytes | 1.9279 | 5.6963 | 3.7683 | 0.019 | 0.135 |
| cd4_t_cells_cd45ro → plasma_cells | 0.2638 | 0.9541 | 0.6904 | 0.021 | 0.142 |
| plasma_cells → cd8_t_cells | 0.1374 | 0.4569 | 0.3196 | 0.023 | 0.150 |
| cd68_macrophages → plasma_cells | -0.0123 | -0.0321 | -0.0198 | 0.025 | 0.156 |
| cd8_t_cells → stroma | 0.9031 | 2.3715 | 1.4685 | 0.027 | 0.156 |

Continued on next page

| Cell type pair | CLR | DII | Effect size | p-value | adjusted p-value |
|---|---|---|---|---|---|
| cd68_macrophages → cd68_cd163_macrophages | -0.0390 | 0.1619 | 0.2008 | 0.027 | 0.156 |
| cd68_cd163_macrophages → granulocytes | 1.9554 | 4.4239 | 2.4685 | 0.030 | 0.164 |
| cd8_t_cells → granulocytes | 0.5026 | 1.5277 | 1.0251 | 0.031 | 0.165 |
| cd68_cd163_macrophages → smooth_muscle | 1.4656 | 3.5419 | 2.0762 | 0.035 | 0.168 |
| tregs → vasculature | -0.0166 | 0.1395 | 0.1561 | 0.036 | 0.168 |
| stroma → smooth_muscle | 1.1695 | 2.3937 | 1.2242 | 0.036 | 0.168 |
| smooth_muscle → plasma_cells | -0.0227 | 0.2698 | 0.2926 | 0.037 | 0.168 |
| tregs → cd4_t_cells_cd45ro | 0.1253 | 0.4506 | 0.3253 | 0.038 | 0.168 |
| cd8_t_cells → b_cells | 2.3809 | 1.3258 | -1.0551 | 0.039 | 0.168 |
| plasma_cells → cd4_t_cells_cd45ro | 0.1134 | 0.4971 | 0.3836 | 0.043 | 0.182 |
| stroma → cd8_t_cells | 1.2573 | 2.5960 | 1.3387 | 0.045 | 0.185 |
| plasma_cells → stroma | 0.1281 | 0.5398 | 0.4117 | 0.046 | 0.186 |
| cd4_t_cells_cd45ro → cd68_cd163_macrophages | 1.0916 | 2.7539 | 1.6623 | 0.052 | 0.198 |
| smooth_muscle → cd4_t_cells_cd45ro | 0.5320 | 1.1519 | 0.6198 | 0.054 | 0.198 |
| stroma → cd4_t_cells_cd45ro | 1.3986 | 2.6925 | 1.2939 | 0.054 | 0.198 |
| smooth_muscle → stroma | 1.0775 | 2.5508 | 1.4733 | 0.055 | 0.198 |
| b_cells → cd68_macrophages | 0.6556 | -0.0325 | -0.6882 | 0.062 | 0.217 |
| cd8_t_cells → cd4_t_cells_cd45ro | 1.5175 | 2.9427 | 1.4252 | 0.064 | 0.220 |
| tregs → cd8_t_cells | 0.2629 | 0.5987 | 0.3358 | 0.068 | 0.223 |
| stroma → vasculature | 1.2654 | 2.2888 | 1.0233 | 0.068 | 0.223 |
| tregs → plasma_cells | -0.0360 | 0.1217 | 0.1577 | 0.071 | 0.227 |
| tregs → tregs | 0.0160 | 0.1183 | 0.1023 | 0.072 | 0.227 |
| granulocytes → cd68_cd163_macrophages | 1.2232 | 3.4755 | 2.2523 | 0.088 | 0.261 |
| cd8_t_cells → tumor_cells | 0.6382 | 1.5702 | 0.9321 | 0.090 | 0.261 |
| tumor_cells → b_cells | 0.6946 | 0.0773 | -0.6173 | 0.096 | 0.261 |
| tregs → cd68_macrophages | -0.0025 | -0.0275 | -0.0251 | 0.096 | 0.261 |
| plasma_cells → smooth_muscle | -0.0356 | 0.0724 | 0.1080 | 0.097 | 0.261 |
| smooth_muscle → cd8_t_cells | 0.6203 | 1.5902 | 0.9699 | 0.097 | 0.261 |

Continued on next page

| Cell type pair | CLR | DII | Effect size | p-value | adjusted p-value |
|---|---|---|---|---|---|
| cd68_cd163_macrophages → plasma_cells | 1.4967 | 3.0173 | 1.5207 | 0.098 | 0.261 |
| vasculature → tregs | 0.0446 | 0.3166 | 0.2720 | 0.098 | 0.261 |
| cd68_macrophages → vasculature | -0.0495 | 0.0103 | 0.0597 | 0.103 | 0.266 |
| cd4_t_cells_cd45ro → stroma | 0.9469 | 1.9338 | 0.9869 | 0.103 | 0.266 |
| smooth_muscle → cd68_cd163_macrophages | 1.2361 | 3.4141 | 2.1780 | 0.106 | 0.267 |
| cd68_macrophages → granulocytes | 0.0624 | 0.1942 | 0.1318 | 0.107 | 0.267 |
| cd68_macrophages → stroma | -0.0177 | 0.2339 | 0.2516 | 0.110 | 0.267 |
| cd68_macrophages → tregs | 0.0007 | -0.0295 | -0.0301 | 0.112 | 0.267 |
| cd68_macrophages → tumor_cells | 0.0337 | 0.1380 | 0.1042 | 0.113 | 0.267 |
| b_cells → vasculature | -0.1779 | 0.1779 | 0.3558 | 0.117 | 0.268 |
| granulocytes → plasma_cells | 0.1627 | 0.5091 | 0.3464 | 0.117 | 0.268 |
| plasma_cells → cd68_cd163_macrophages | 0.2602 | 0.6486 | 0.3884 | 0.127 | 0.281 |
| b_cells → granulocytes | -0.1008 | 0.2613 | 0.3621 | 0.127 | 0.281 |
| granulocytes → stroma | 0.9707 | 2.3589 | 1.3882 | 0.130 | 0.281 |
| tregs → granulocytes | 0.0780 | 0.2707 | 0.1927 | 0.131 | 0.281 |
| cd68_cd163_macrophages → tumor_cells | 2.8960 | 4.1929 | 1.2969 | 0.135 | 0.285 |
| vasculature → plasma_cells | 0.2173 | 0.6360 | 0.4187 | 0.140 | 0.290 |
| vasculature → stroma | 1.0629 | 1.8770 | 0.8141 | 0.141 | 0.290 |
| tregs → smooth_muscle | 0.0063 | 0.2354 | 0.2291 | 0.147 | 0.298 |
| b_cells → plasma_cells | -0.1005 | 0.1791 | 0.2796 | 0.153 | 0.301 |
| smooth_muscle → tumor_cells | 0.5549 | 1.2395 | 0.6846 | 0.154 | 0.301 |
| cd68_macrophages → b_cells | 0.0577 | -0.0185 | -0.0761 | 0.155 | 0.301 |
| tregs → tumor_cells | -0.0189 | 0.1517 | 0.1706 | 0.163 | 0.313 |
| cd4_t_cells_cd45ro → granulocytes | 0.7315 | 1.1249 | 0.3934 | 0.169 | 0.321 |
| granulocytes → cd8_t_cells | 0.8982 | 1.5810 | 0.6828 | 0.185 | 0.347 |
| smooth_muscle → tregs | 0.1473 | 0.6421 | 0.4948 | 0.192 | 0.355 |

Continued on next page

| Cell type pair | CLR | DII | Effect size | p-value | adjusted p-value |
|---|---|---|---|---|---|
| cd4_t_cells_cd45ro → vasculature | 0.6152 | 1.0019 | 0.3867 | 0.216 | 0.394 |
| plasma_cells → plasma_cells | 0.4188 | 0.9474 | 0.5286 | 0.221 | 0.398 |
| tregs → b_cells | 0.1793 | 0.3481 | 0.1688 | 0.231 | 0.411 |
| cd68_macrophages → cd68_macrophages | -0.0013 | -0.0090 | -0.0076 | 0.237 | 0.416 |
| stroma → granulocytes | 0.9975 | 1.7349 | 0.7374 | 0.244 | 0.424 |
| stroma → tregs | 0.5404 | 1.0487 | 0.5083 | 0.256 | 0.439 |
| plasma_cells → cd68_macrophages | -0.0111 | -0.0237 | -0.0126 | 0.259 | 0.439 |
| plasma_cells → vasculature | 0.1174 | 0.2745 | 0.1571 | 0.269 | 0.450 |
| cd8_t_cells → cd8_t_cells | 1.9722 | 2.7653 | 0.7931 | 0.277 | 0.454 |
| vasculature → cd68_cd163_macrophages | 0.9998 | 1.5933 | 0.5936 | 0.278 | 0.454 |
| granulocytes → smooth_muscle | 0.3037 | 1.5129 | 1.2093 | 0.302 | 0.488 |
| cd68_cd163_macrophages → stroma | 4.4427 | 5.8631 | 1.4204 | 0.306 | 0.490 |
| tumor_cells → vasculature | 0.6008 | 0.2929 | -0.3079 | 0.324 | 0.509 |
| cd68_cd163_macrophages → cd68_macrophages | 0.6434 | 1.0807 | 0.4373 | 0.325 | 0.509 |
| vasculature → b_cells | 0.6827 | 0.4406 | -0.2421 | 0.338 | 0.523 |
| vasculature → cd4_t_cells_cd45ro | 0.7993 | 1.1131 | 0.3137 | 0.344 | 0.528 |
| vasculature → vasculature | 3.0572 | 3.9362 | 0.8790 | 0.352 | 0.534 |
| granulocytes → cd68_macrophages | 0.3844 | 0.6483 | 0.2638 | 0.366 | 0.550 |
| granulocytes → vasculature | 1.0023 | 1.6729 | 0.6706 | 0.374 | 0.556 |
| stroma → cd68_macrophages | 0.3933 | 0.6651 | 0.2718 | 0.408 | 0.592 |
| cd4_t_cells_cd45ro → smooth_muscle | 0.5148 | 0.8261 | 0.3114 | 0.409 | 0.592 |
| stroma → b_cells | 1.0701 | 0.7679 | -0.3022 | 0.411 | 0.592 |
| cd8_t_cells → tregs | 0.8538 | 1.1391 | 0.2853 | 0.442 | 0.617 |
| stroma → tumor_cells | 1.8813 | 2.3820 | 0.5007 | 0.443 | 0.617 |
| cd68_cd163_macrophages → vasculature | 2.7715 | 3.4681 | 0.6967 | 0.445 | 0.617 |
| cd68_macrophages → cd4_t_cells_cd45ro | 0.0531 | 0.1108 | 0.0577 | 0.446 | 0.617 |

Continued on next page

| Cell type pair | CLR | DII | Effect size | p-value | adjusted p-value |
|---|---|---|---|---|---|
| plasma_cells → tumor_cells | 0.1293 | 0.2154 | 0.0861 | 0.478 | 0.655 |
| b_cells → tumor_cells | 0.3485 | 0.0650 | -0.2835 | 0.485 | 0.659 |
| vasculature → cd68_macrophages | -0.0446 | -0.0185 | 0.0261 | 0.492 | 0.662 |
| granulocytes → tregs | 0.6677 | 1.2482 | 0.5804 | 0.499 | 0.665 |
| b_cells → smooth_muscle | -0.0907 | 0.0442 | 0.1349 | 0.518 | 0.684 |
| tumor_cells → cd68_cd163_macrophages | 1.5854 | 1.1905 | -0.3949 | 0.541 | 0.704 |
| tumor_cells → cd4_t_cells_cd45ro | 0.7995 | 0.2726 | -0.5270 | 0.544 | 0.704 |
| smooth_muscle → b_cells | 0.0294 | 0.1126 | 0.0832 | 0.548 | 0.704 |
| cd4_t_cells_cd45ro → cd4_t_cells_cd45ro | 2.3927 | 1.9139 | -0.4788 | 0.561 | 0.714 |
| tumor_cells → plasma_cells | 0.2745 | 0.0895 | -0.1850 | 0.575 | 0.721 |
| plasma_cells → tregs | -0.0228 | -0.0058 | 0.0170 | 0.580 | 0.721 |
| cd68_cd163_macrophages → b_cells | 1.8564 | 2.2118 | 0.3554 | 0.581 | 0.721 |
| cd68_macrophages → smooth_muscle | -0.0162 | 0.0164 | 0.0326 | 0.597 | 0.735 |
| smooth_muscle → granulocytes | 0.9881 | 1.6802 | 0.6921 | 0.620 | 0.748 |
| plasma_cells → b_cells | 0.0850 | 0.1363 | 0.0513 | 0.622 | 0.748 |
| stroma → stroma | 2.6215 | 3.0533 | 0.4317 | 0.623 | 0.748 |
| cd8_t_cells → vasculature | 0.7121 | 0.8602 | 0.1481 | 0.634 | 0.752 |
| vasculature → smooth_muscle | 1.6168 | 1.3348 | -0.2820 | 0.637 | 0.752 |
| tumor_cells → smooth_muscle | 0.2439 | 0.0506 | -0.1933 | 0.680 | 0.796 |
| vasculature → cd8_t_cells | 0.8742 | 0.7584 | -0.1158 | 0.715 | 0.831 |
| cd4_t_cells_cd45ro → cd68_macrophages | 0.1951 | 0.2569 | 0.0618 | 0.743 | 0.843 |
| tregs → stroma | 0.2848 | 0.3406 | 0.0558 | 0.743 | 0.843 |
| tumor_cells → cd8_t_cells | 0.5623 | 0.7065 | 0.1442 | 0.752 | 0.843 |
| smooth_muscle → cd68_macrophages | -0.0145 | -0.0383 | -0.0238 | 0.755 | 0.843 |
| cd4_t_cells_cd45ro → tumor_cells | 1.0524 | 1.1909 | 0.1385 | 0.757 | 0.843 |
| granulocytes → b_cells | 0.9734 | 1.2347 | 0.2614 | 0.765 | 0.843 |
| smooth_muscle → vasculature | 1.5689 | 1.7666 | 0.1977 | 0.767 | 0.843 |

Continued on next page

| Cell type pair | CLR | DII | Effect size | p-value | adjusted p-value |
|---|---|---|---|---|---|
| plasma_cells → granulocytes | 0.0388 | 0.0594 | 0.0206 | 0.798 | 0.871 |
| tumor_cells → cd68_macrophages | 0.2884 | 0.2462 | -0.0421 | 0.830 | 0.892 |
| cd8_t_cells → cd68_macrophages | 0.1706 | 0.2047 | 0.0341 | 0.830 | 0.892 |
| vasculature → tumor_cells | 0.3142 | 0.3591 | 0.0449 | 0.851 | 0.897 |
| cd4_t_cells_cd45ro → tregs | 0.8603 | 0.7943 | -0.0660 | 0.852 | 0.897 |
| tumor_cells → tregs | 0.0072 | 0.1015 | 0.0943 | 0.853 | 0.897 |
| cd4_t_cells_cd45ro → cd8_t_cells | 2.7711 | 2.6284 | -0.1426 | 0.874 | 0.908 |
| smooth_muscle → smooth_muscle | 4.4477 | 4.7447 | 0.2970 | 0.877 | 0.908 |
| vasculature → granulocytes | 1.1917 | 1.1305 | -0.0612 | 0.902 | 0.928 |
| cd68_macrophages → cd8_t_cells | 0.0246 | 0.0295 | 0.0048 | 0.948 | 0.967 |
| stroma → cd68_cd163_macrophages | 3.6988 | 3.7617 | 0.0629 | 0.955 | 0.967 |
| tumor_cells → stroma | 1.3315 | 1.3669 | 0.0354 | 0.960 | 0.967 |
| b_cells → stroma | 0.1632 | 0.1822 | 0.0189 | 0.971 | 0.971 |

## S8.4 CRC runtime

Table S2: Runtime for the primary CRC PANORAMIC analysis on Stanford Sherlock HPC using 10 CPUs.

| Analysis step | Runtime (min) |
|---|---|
| Data preparation | 0.92 |
| Spatial statistic and bootstrap estimation | 1.37 |
| Multilevel meta-analysis | 3.67 |
| Total | 5.96 |

## S8.5 CRC radius stability

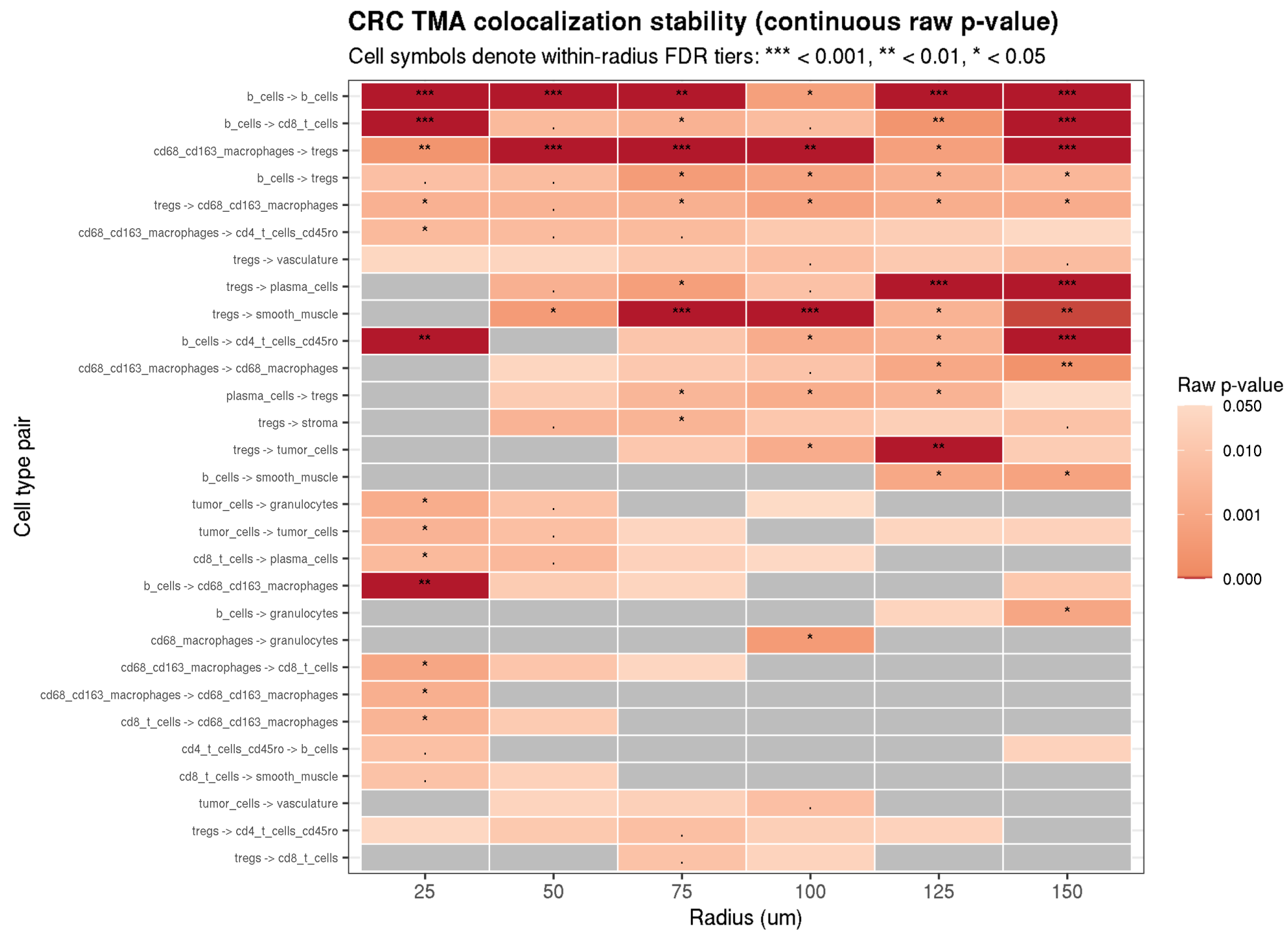


Figure S4: Stability of PANORAMIC results across different radii. The plotted cell pairs are those with results at or near statistical significance at any of the radii. Gray boxes indicate raw p-values $\geq 0.05$, while shades of red indicate raw p-values achieving statistical significance $p < 0.05$. Asterisks indicate the p-values that retain statistical significance after performing Benjamini-Hochberg multiple testing correction.

## S8.6 CRC spicyR benchmark

Table S3: CRC TMA spicyR results (benchmark context).

| Cell type pair | Effect size | p-value | adjusted p-value |
|---|---:|---:|---:|
| cd8_t_cells → smooth_muscle | 251.1721 | 0.000 | 0.025 |
| smooth_muscle → cd8_t_cells | 249.3169 | 0.000 | 0.025 |
| tregs → tregs | 624.6480 | 0.002 | 0.076 |
| cd68_cd163_macrophages → b_cells | 245.7006 | 0.006 | 0.121 |
| cd68_cd163_macrophages → tumor_cells | -183.0408 | 0.006 | 0.121 |
| b_cells → cd68_cd163_macrophages | 244.7538 | 0.006 | 0.121 |
| tumor_cells → cd68_cd163_macrophages | -176.1195 | 0.008 | 0.144 |
| cd8_t_cells → granulocytes | 157.4626 | 0.056 | 0.705 |

Continued on next page

| Cell type pair | Effect size | p-value | adjusted p-value |
|---|---|---|---|
| granulocytes → cd8_t_cells | 157.2493 | 0.057 | 0.705 |
| b_cells → smooth_muscle | 177.9288 | 0.063 | 0.705 |
| smooth_muscle → b_cells | 177.1203 | 0.064 | 0.705 |
| granulocytes → b_cells | 156.4979 | 0.078 | 0.781 |
| b_cells → granulocytes | 150.0650 | 0.091 | 0.781 |
| cd4_t_cells_cd45ro → plasma_cells | 160.0683 | 0.094 | 0.781 |
| smooth_muscle → granulocytes | -242.1919 | 0.104 | 0.781 |
| plasma_cells → cd4_t_cells_cd45ro | 153.5992 | 0.114 | 0.781 |
| stroma → tumor_cells | -140.1818 | 0.124 | 0.781 |
| granulocytes → smooth_muscle | -220.6485 | 0.127 | 0.781 |
| smooth_muscle → smooth_muscle | -507.0795 | 0.155 | 0.781 |
| tumor_cells → stroma | -128.0081 | 0.158 | 0.781 |
| tumor_cells → vasculature | -214.8372 | 0.164 | 0.781 |
| vasculature → smooth_muscle | -172.0975 | 0.166 | 0.781 |
| plasma_cells → tregs | 280.9786 | 0.167 | 0.781 |
| smooth_muscle → vasculature | -170.9496 | 0.167 | 0.781 |
| granulocytes → vasculature | -225.4646 | 0.168 | 0.781 |
| vasculature → tumor_cells | -211.4625 | 0.171 | 0.781 |
| vasculature → granulocytes | -232.0506 | 0.177 | 0.781 |
| tregs → plasma_cells | 270.3349 | 0.181 | 0.781 |
| cd68_cd163_macrophages → smooth_muscle | 102.3360 | 0.212 | 0.781 |
| cd8_t_cells → cd8_t_cells | -392.7014 | 0.224 | 0.781 |
| smooth_muscle → cd68_cd163_macrophages | 99.4946 | 0.225 | 0.781 |
| plasma_cells → cd68_cd163_macrophages | -145.0082 | 0.236 | 0.781 |
| cd8_t_cells → cd4_t_cells_cd45ro | -161.4450 | 0.238 | 0.781 |
| vasculature → cd68_cd163_macrophages | -96.3026 | 0.245 | 0.781 |

Continued on next page

| Cell type pair | Effect size | p-value | adjusted p-value |
|---|---|---|---|
| cd4_t_cells_cd45ro → cd8_t_cells | -158.2990 | 0.247 | 0.781 |
| cd68_cd163_macrophages → plasma_cells | -141.3895 | 0.250 | 0.781 |
| cd68_cd163_macrophages → vasculature | -95.8274 | 0.254 | 0.781 |
| plasma_cells → tumor_cells | -235.4651 | 0.256 | 0.781 |
| b_cells → vasculature | 114.8588 | 0.257 | 0.781 |
| vasculature → b_cells | 113.4083 | 0.261 | 0.781 |
| tumor_cells → plasma_cells | -231.1822 | 0.265 | 0.781 |
| tumor_cells → smooth_muscle | -87.9551 | 0.297 | 0.855 |
| smooth_muscle → tumor_cells | -86.5623 | 0.304 | 0.855 |
| b_cells → plasma_cells | 121.6284 | 0.315 | 0.863 |
| plasma_cells → b_cells | 117.1395 | 0.336 | 0.863 |
| b_cells → b_cells | -213.4992 | 0.353 | 0.863 |
| tregs → cd68_cd163_macrophages | 95.8850 | 0.398 | 0.863 |
| cd68_cd163_macrophages → tregs | 94.4650 | 0.404 | 0.863 |
| cd8_t_cells → tregs | -373.6227 | 0.409 | 0.863 |
| tregs → cd8_t_cells | -347.9804 | 0.415 | 0.863 |
| tumor_cells → tregs | 91.3017 | 0.416 | 0.863 |
| stroma → b_cells | 80.6026 | 0.416 | 0.863 |
| tumor_cells → b_cells | -74.4638 | 0.417 | 0.863 |
| tregs → smooth_muscle | 170.3143 | 0.419 | 0.863 |
| smooth_muscle → tregs | 168.8990 | 0.422 | 0.863 |
| stroma → cd68_cd163_macrophages | 38.7122 | 0.425 | 0.863 |
| cd68_cd163_macrophages → stroma | 38.5283 | 0.433 | 0.863 |
| b_cells → stroma | 75.2188 | 0.447 | 0.863 |
| tumor_cells → tumor_cells | 172.4588 | 0.448 | 0.863 |
| b_cells → tumor_cells | -69.6674 | 0.450 | 0.863 |
| tregs → tumor_cells | 84.3848 | 0.452 | 0.863 |
| cd8_t_cells → tumor_cells | -46.3800 | 0.452 | 0.863 |
| cd4_t_cells_cd45ro → stroma | 60.2268 | 0.462 | 0.863 |

Continued on next page

| Cell type pair | Effect size | p-value | adjusted p-value |
|---|---|---|---|
| plasma_cells → smooth_muscle | -149.2790 | 0.468 | 0.863 |
| smooth_muscle → plasma_cells | -148.0778 | 0.472 | 0.863 |
| granulocytes → granulocytes | -240.2355 | 0.474 | 0.863 |
| stroma → plasma_cells | 75.8563 | 0.489 | 0.863 |
| tumor_cells → cd8_t_cells | -42.8934 | 0.492 | 0.863 |
| cd68_cd163_macrophages → granulocytes | -69.7031 | 0.492 | 0.863 |
| granulocytes → cd68_cd163_macrophages | -59.5111 | 0.532 | 0.875 |
| cd68_cd163_macrophages → cd4_t_cells_cd45ro | 25.5254 | 0.554 | 0.875 |
| vasculature → stroma | -139.0217 | 0.560 | 0.875 |
| cd4_t_cells_cd45ro → vasculature | 51.6935 | 0.564 | 0.875 |
| stroma → vasculature | -137.1140 | 0.565 | 0.875 |
| cd4_t_cells_cd45ro → cd4_t_cells_cd45ro | -66.9544 | 0.568 | 0.875 |
| cd68_cd163_macrophages → cd68_cd163_macrophages | -34.9940 | 0.576 | 0.875 |
| vasculature → cd4_t_cells_cd45ro | 49.8473 | 0.583 | 0.875 |
| cd4_t_cells_cd45ro → tregs | 68.1958 | 0.584 | 0.875 |
| tregs → cd4_t_cells_cd45ro | 67.6346 | 0.586 | 0.875 |
| plasma_cells → granulocytes | -70.2675 | 0.593 | 0.875 |
| plasma_cells → stroma | 59.6292 | 0.598 | 0.875 |
| vasculature → tregs | -138.4905 | 0.598 | 0.875 |
| cd4_t_cells_cd45ro → granulocytes | -79.5047 | 0.600 | 0.875 |
| tregs → vasculature | -125.1239 | 0.619 | 0.886 |
| granulocytes → cd4_t_cells_cd45ro | -75.0380 | 0.622 | 0.886 |
| cd4_t_cells_cd45ro → tumor_cells | -43.4588 | 0.647 | 0.911 |
| vasculature → cd8_t_cells | -60.0413 | 0.685 | 0.934 |
| cd8_t_cells → vasculature | -57.0689 | 0.697 | 0.934 |
| granulocytes → plasma_cells | -44.4338 | 0.705 | 0.934 |

Continued on next page

| Cell type pair | Effect size | p-value | adjusted p-value |
|---|---|---|---|
| tregs → b_cells | -66.9695 | 0.716 | 0.934 |
| b_cells → tregs | -65.8548 | 0.720 | 0.934 |
| stroma → granulocytes | 34.4677 | 0.733 | 0.934 |
| tumor_cells → cd4_t_cells_cd45ro | -27.9978 | 0.747 | 0.934 |
| smooth_muscle → cd4_t_cells_cd45ro | 29.3268 | 0.758 | 0.934 |
| b_cells → cd4_t_cells_cd45ro | -41.8900 | 0.762 | 0.934 |
| stroma → stroma | 31.7435 | 0.765 | 0.934 |
| stroma → tregs | 35.5145 | 0.768 | 0.934 |
| tregs → stroma | 35.4231 | 0.768 | 0.934 |
| plasma_cells → vasculature | -31.7610 | 0.777 | 0.934 |
| cd4_t_cells_cd45ro → smooth_muscle | 25.4418 | 0.789 | 0.934 |
| plasma_cells → plasma_cells | 66.3587 | 0.789 | 0.934 |
| stroma → cd4_t_cells_cd45ro | 27.9173 | 0.790 | 0.934 |
| cd4_t_cells_cd45ro → b_cells | -35.5157 | 0.797 | 0.934 |
| smooth_muscle → stroma | -20.1175 | 0.803 | 0.934 |
| vasculature → plasma_cells | -21.6034 | 0.846 | 0.957 |
| tregs → granulocytes | 47.7693 | 0.863 | 0.957 |
| cd8_t_cells → cd68_cd163_macrophages | 10.4699 | 0.868 | 0.957 |
| granulocytes → tregs | 45.2522 | 0.870 | 0.957 |
| cd68_cd163_macrophages → cd8_t_cells | 10.1707 | 0.871 | 0.957 |
| granulocytes → stroma | 16.7709 | 0.874 | 0.957 |
| cd4_t_cells_cd45ro → cd68_cd163_macrophages | -8.4006 | 0.893 | 0.957 |
| cd8_t_cells → plasma_cells | 12.8735 | 0.897 | 0.957 |
| tumor_cells → granulocytes | -12.7706 | 0.901 | 0.957 |
| plasma_cells → cd8_t_cells | 12.1952 | 0.902 | 0.957 |
| granulocytes → tumor_cells | -8.2974 | 0.937 | 0.962 |
| b_cells → cd8_t_cells | -9.3289 | 0.942 | 0.962 |
| vasculature → vasculature | -17.6214 | 0.944 | 0.962 |
| stroma → cd8_t_cells | -3.9883 | 0.951 | 0.962 |

Continued on next page

| Cell type pair | Effect size | p-value | adjusted p-value |
|---|---|---|---|
| cd8_t_cells → b_cells | -7.0978 | 0.956 | 0.962 |
| stroma → smooth_muscle | -4.1175 | 0.958 | 0.962 |
| cd8_t_cells → stroma | -3.0497 | 0.962 | 0.962 |

## S8.7 CRC Welch *t*-test benchmark

Table S4: CRC TMA t-test results (benchmark context).

| Cell type pair | CLR | DII | Effect size | p-value | adjusted p-value |
|---|---|---|---|---|---|
| cd68_cd163_macrophages → tregs | 0.3888 | 2.9327 | 2.5439 | 0.000 | 0.026 |
| cd68_cd163_macrophages → cd8_t_cells | 3.3958 | 7.3109 | 3.9151 | 0.001 | 0.086 |
| cd68_cd163_macrophages → cd4_t_cells_cd45ro | 3.0112 | 5.6315 | 2.6203 | 0.004 | 0.116 |
| cd8_t_cells → cd68_cd163_macrophages | 1.1666 | 4.2916 | 3.1249 | 0.004 | 0.116 |
| cd68_cd163_macrophages → cd68_cd163_macrophages | 4.3849 | 8.3798 | 3.9949 | 0.005 | 0.116 |
| cd8_t_cells → plasma_cells | 0.2189 | 1.5812 | 1.3623 | 0.007 | 0.116 |
| tumor_cells → tumor_cells | 3.5428 | 11.1261 | 7.5833 | 0.007 | 0.116 |
| cd8_t_cells → smooth_muscle | 0.4515 | 1.7224 | 1.2709 | 0.007 | 0.116 |
| b_cells → b_cells | 6.1335 | 0.5409 | -5.5926 | 0.007 | 0.116 |
| cd8_t_cells → stroma | 0.9618 | 2.6970 | 1.7352 | 0.018 | 0.263 |
| tregs → vasculature | 0.0189 | 0.2538 | 0.2349 | 0.021 | 0.263 |
| granulocytes → granulocytes | 2.1523 | 6.0970 | 3.9447 | 0.023 | 0.263 |
| cd4_t_cells_cd45ro → plasma_cells | 0.3732 | 1.2305 | 0.8572 | 0.024 | 0.263 |
| cd8_t_cells → cd4_t_cells_cd45ro | 1.5184 | 3.4110 | 1.8927 | 0.029 | 0.266 |
| stroma → plasma_cells | 0.5656 | 1.7618 | 1.1962 | 0.030 | 0.266 |
| b_cells → cd8_t_cells | 3.1945 | 0.5928 | -2.6017 | 0.032 | 0.266 |
| cd8_t_cells → b_cells | 2.8946 | 1.5178 | -1.3768 | 0.035 | 0.266 |
| granulocytes → cd4_t_cells_cd45ro | 0.7680 | 2.0269 | 1.2589 | 0.036 | 0.266 |
| tregs → cd68_cd163_macrophages | 0.1571 | 0.7627 | 0.6055 | 0.037 | 0.266 |
| tregs → cd8_t_cells | 0.3289 | 0.7441 | 0.4152 | 0.038 | 0.266 |
| cd8_t_cells → granulocytes | 0.5852 | 1.7887 | 1.2035 | 0.039 | 0.266 |
| stroma → cd8_t_cells | 1.3573 | 2.8286 | 1.4713 | 0.042 | 0.274 |
| cd4_t_cells_cd45ro → b_cells | 3.9396 | 1.4057 | -2.5339 | 0.047 | 0.282 |

Continued on next page

| Cell type pair | CLR | DII | Effect size | p-value | adjusted p-value |
|---|---|---|---|---|---|
| cd68_cd163_macrophages → smooth_muscle | 1.7049 | 3.8653 | 2.1604 | 0.047 | 0.282 |
| tumor_cells → b_cells | 1.3519 | -0.0227 | -1.3745 | 0.056 | 0.303 |
| stroma → smooth_muscle | 1.4441 | 2.5866 | 1.1424 | 0.061 | 0.303 |
| stroma → cd4_t_cells_cd45ro | 1.5898 | 2.9742 | 1.3844 | 0.061 | 0.303 |
| tregs → granulocytes | 0.1280 | 0.4380 | 0.3100 | 0.062 | 0.303 |
| tumor_cells → vasculature | 1.0346 | 0.1851 | -0.8494 | 0.063 | 0.303 |
| stroma → vasculature | 1.4733 | 2.5787 | 1.1054 | 0.063 | 0.303 |
| plasma_cells → cd8_t_cells | 0.3691 | 1.0801 | 0.7110 | 0.068 | 0.308 |
| cd4_t_cells_cd45ro → cd68_cd163_macrophages | 1.2107 | 2.9821 | 1.7714 | 0.069 | 0.308 |
| b_cells → cd68_cd163_macrophages | -3.8839 | 0.4900 | 4.3739 | 0.076 | 0.330 |
| tregs → tumor_cells | 0.0606 | 0.3666 | 0.3059 | 0.083 | 0.330 |
| vasculature → tregs | 0.2061 | 0.6673 | 0.4612 | 0.084 | 0.330 |
| granulocytes → cd68_cd163_macrophages | 1.4476 | 3.9043 | 2.4568 | 0.085 | 0.330 |
| plasma_cells → tregs | 0.0815 | 0.4898 | 0.4083 | 0.085 | 0.330 |
| stroma → tregs | 0.5841 | 1.3336 | 0.7495 | 0.088 | 0.330 |
| cd68_cd163_macrophages → granulocytes | 2.1650 | 4.3064 | 2.1414 | 0.089 | 0.330 |
| smooth_muscle → plasma_cells | 0.2229 | 0.6059 | 0.3830 | 0.094 | 0.340 |
| smooth_muscle → cd68_cd163_macrophages | 1.4215 | 3.6917 | 2.2702 | 0.099 | 0.346 |
| smooth_muscle → b_cells | 0.1846 | 0.9048 | 0.7202 | 0.105 | 0.348 |
| granulocytes → stroma | 1.1343 | 2.8188 | 1.6845 | 0.107 | 0.348 |
| cd68_cd163_macrophages → plasma_cells | 1.6140 | 3.2774 | 1.6634 | 0.108 | 0.348 |
| plasma_cells → cd4_t_cells_cd45ro | 0.3336 | 1.0535 | 0.7199 | 0.115 | 0.348 |
| b_cells → tregs | 1.6558 | 0.4227 | -1.2331 | 0.115 | 0.348 |
| tregs → plasma_cells | 0.0308 | 0.2313 | 0.2005 | 0.117 | 0.348 |
| vasculature → stroma | 1.2066 | 2.1577 | 0.9511 | 0.119 | 0.348 |
| plasma_cells → vasculature | 0.3049 | 1.0625 | 0.7576 | 0.120 | 0.348 |
| cd4_t_cells_cd45ro → stroma | 1.0452 | 2.0789 | 1.0337 | 0.123 | 0.348 |
| cd8_t_cells → tumor_cells | 0.9411 | 2.0705 | 1.1294 | 0.125 | 0.348 |

Continued on next page

| Cell type pair | CLR | DII | Effect size | p-value | adjusted p-value |
|---|---|---|---|---|---|
| cd68_macrophages → cd4_t_cells_cd45ro | 0.0779 | 0.2699 | 0.1921 | 0.127 | 0.348 |
| plasma_cells → stroma | 0.2741 | 1.3100 | 1.0359 | 0.128 | 0.348 |
| cd68_macrophages → smooth_muscle | -0.0351 | 0.1531 | 0.1882 | 0.143 | 0.382 |
| cd8_t_cells → cd8_t_cells | 2.1039 | 3.2119 | 1.1080 | 0.165 | 0.431 |
| tregs → b_cells | 0.2399 | 0.5160 | 0.2761 | 0.169 | 0.431 |
| smooth_muscle → cd4_t_cells_cd45ro | 0.9365 | 1.4208 | 0.4844 | 0.172 | 0.431 |
| smooth_muscle → tumor_cells | 0.8319 | 1.7085 | 0.8766 | 0.174 | 0.431 |
| b_cells → plasma_cells | -0.4199 | 0.3782 | 0.7981 | 0.178 | 0.435 |
| granulocytes → tumor_cells | 1.2767 | 2.3389 | 1.0622 | 0.184 | 0.436 |
| smooth_muscle → stroma | 1.5022 | 2.9195 | 1.4173 | 0.185 | 0.436 |
| plasma_cells → plasma_cells | 0.5207 | 1.4604 | 0.9396 | 0.190 | 0.439 |
| cd68_macrophages → granulocytes | 0.3277 | 0.7876 | 0.4599 | 0.197 | 0.439 |
| granulocytes → smooth_muscle | 0.3092 | 1.8484 | 1.5391 | 0.198 | 0.439 |
| vasculature → plasma_cells | 0.3559 | 0.8197 | 0.4638 | 0.199 | 0.439 |
| tumor_cells → cd68_cd163_macrophages | 2.3118 | 1.0797 | -1.2320 | 0.204 | 0.439 |
| smooth_muscle → tregs | 0.3351 | 1.0185 | 0.6834 | 0.204 | 0.439 |
| vasculature → cd68_macrophages | 0.0088 | 0.1998 | 0.1910 | 0.214 | 0.453 |
| granulocytes → plasma_cells | 0.4356 | 1.0530 | 0.6174 | 0.230 | 0.473 |
| tumor_cells → granulocytes | 0.8646 | 1.9560 | 1.0915 | 0.230 | 0.473 |
| smooth_muscle → cd8_t_cells | 1.0817 | 2.0250 | 0.9433 | 0.238 | 0.479 |
| b_cells → smooth_muscle | -2.6542 | 0.1540 | 2.8082 | 0.240 | 0.479 |
| granulocytes → vasculature | 1.1221 | 2.1126 | 0.9905 | 0.261 | 0.508 |
| vasculature → cd68_cd163_macrophages | 1.1540 | 1.8157 | 0.6617 | 0.266 | 0.508 |
| plasma_cells → cd68_cd163_macrophages | 0.6364 | 1.3597 | 0.7233 | 0.266 | 0.508 |
| cd8_t_cells → cd68_macrophages | 0.2586 | 0.5674 | 0.3088 | 0.268 | 0.508 |
| stroma → granulocytes | 1.1106 | 1.8341 | 0.7235 | 0.288 | 0.539 |

Continued on next page

| Cell type pair | CLR | DII | Effect size | p-value | adjusted p-value |
|---|---|---|---|---|---|
| cd68_cd163_macrophages → cd68_macrophages | 0.8619 | 1.5683 | 0.7063 | 0.299 | 0.552 |
| cd68_cd163_macrophages → stroma | 4.7266 | 6.0443 | 1.3177 | 0.317 | 0.569 |
| plasma_cells → tumor_cells | 0.4013 | 0.6695 | 0.2682 | 0.322 | 0.569 |
| b_cells → granulocytes | -1.1695 | 0.3779 | 1.5473 | 0.326 | 0.569 |
| b_cells → cd4_t_cells_cd45ro | 2.1926 | 0.6334 | -1.5591 | 0.327 | 0.569 |
| cd4_t_cells_cd45ro → tumor_cells | 1.9195 | 1.4047 | -0.5149 | 0.328 | 0.569 |
| granulocytes → cd68_macrophages | 0.8021 | 1.7159 | 0.9138 | 0.342 | 0.587 |
| cd68_cd163_macrophages → b_cells | 1.9624 | 2.5567 | 0.5943 | 0.348 | 0.588 |
| cd8_t_cells → tregs | 1.0028 | 1.4404 | 0.4375 | 0.351 | 0.588 |
| vasculature → b_cells | 1.0725 | 0.6654 | -0.4071 | 0.359 | 0.592 |
| stroma → cd68_macrophages | 0.6563 | 1.1382 | 0.4819 | 0.362 | 0.592 |
| granulocytes → cd8_t_cells | 1.2963 | 2.0087 | 0.7124 | 0.368 | 0.595 |
| granulocytes → tregs | 0.8803 | 1.7645 | 0.8843 | 0.388 | 0.621 |
| cd68_macrophages → tumor_cells | 0.2900 | 0.4739 | 0.1839 | 0.394 | 0.623 |
| cd4_t_cells_cd45ro → cd4_t_cells_cd45ro | 3.1861 | 2.2632 | -0.9229 | 0.398 | 0.623 |
| cd4_t_cells_cd45ro → tregs | 1.4390 | 0.9289 | -0.5101 | 0.407 | 0.631 |
| tumor_cells → plasma_cells | 0.4247 | 0.0314 | -0.3933 | 0.434 | 0.659 |
| plasma_cells → smooth_muscle | 0.0503 | 0.1416 | 0.0914 | 0.435 | 0.659 |
| vasculature → smooth_muscle | 2.3117 | 1.5219 | -0.7898 | 0.445 | 0.661 |
| b_cells → vasculature | -1.1138 | 0.3556 | 1.4694 | 0.446 | 0.661 |
| tregs → tregs | 0.1542 | 0.2722 | 0.1180 | 0.450 | 0.661 |
| stroma → b_cells | 1.3120 | 0.9925 | -0.3195 | 0.459 | 0.668 |
| cd8_t_cells → vasculature | 0.8764 | 1.1320 | 0.2556 | 0.470 | 0.675 |
| cd68_cd163_macrophages → vasculature | 3.0507 | 3.7147 | 0.6640 | 0.478 | 0.675 |
| cd68_macrophages → tregs | 0.0717 | 0.1418 | 0.0700 | 0.486 | 0.675 |
| cd68_macrophages → vasculature | 0.0547 | 0.1444 | 0.0897 | 0.487 | 0.675 |

Continued on next page

| Cell type pair | CLR | DII | Effect size | p-value | adjusted p-value |
|---|---|---|---|---|---|
| plasma_cells → granulocytes | 0.4269 | 0.7089 | 0.2820 | 0.488 | 0.675 |
| cd68_cd163_macrophages → tumor_cells | 3.6438 | 4.3608 | 0.7171 | 0.493 | 0.676 |
| b_cells → tumor_cells | -0.3783 | 0.3109 | 0.6892 | 0.501 | 0.681 |
| cd4_t_cells_cd45ro → cd68_macrophages | 0.1515 | 0.4425 | 0.2910 | 0.514 | 0.692 |
| smooth_muscle → granulocytes | 1.0884 | 1.8825 | 0.7941 | 0.537 | 0.710 |
| vasculature → vasculature | 3.4675 | 4.1105 | 0.6431 | 0.537 | 0.710 |
| cd68_macrophages → cd68_cd163_macrophages | 0.3382 | 0.6162 | 0.2780 | 0.579 | 0.758 |
| tregs → cd4_t_cells_cd45ro | 0.4524 | 0.6259 | 0.1734 | 0.604 | 0.758 |
| plasma_cells → b_cells | 0.4271 | 0.6176 | 0.1905 | 0.609 | 0.758 |
| tumor_cells → tregs | -0.0986 | 0.1941 | 0.2928 | 0.615 | 0.758 |
| cd68_macrophages → plasma_cells | 0.2110 | 0.1246 | -0.0864 | 0.620 | 0.758 |
| tregs → smooth_muscle | 0.2713 | 0.4470 | 0.1757 | 0.628 | 0.758 |
| cd68_macrophages → stroma | 0.3265 | 0.5123 | 0.1858 | 0.629 | 0.758 |
| tregs → stroma | 0.3807 | 0.4753 | 0.0946 | 0.632 | 0.758 |
| smooth_muscle → vasculature | 2.4722 | 1.9863 | -0.4859 | 0.633 | 0.758 |
| vasculature → granulocytes | 1.5991 | 1.3185 | -0.2806 | 0.635 | 0.758 |
| cd4_t_cells_cd45ro → smooth_muscle | 0.5805 | 0.8273 | 0.2468 | 0.642 | 0.758 |
| cd4_t_cells_cd45ro → granulocytes | 1.0906 | 1.3339 | 0.2434 | 0.642 | 0.758 |
| vasculature → cd4_t_cells_cd45ro | 1.6034 | 1.3443 | -0.2591 | 0.645 | 0.758 |
| cd4_t_cells_cd45ro → cd8_t_cells | 3.3961 | 2.8713 | -0.5248 | 0.648 | 0.758 |
| vasculature → tumor_cells | 0.6965 | 0.5671 | -0.1294 | 0.665 | 0.771 |
| vasculature → cd8_t_cells | 1.2024 | 1.0405 | -0.1620 | 0.669 | 0.771 |
| cd68_macrophages → b_cells | 0.0733 | 0.1085 | 0.0352 | 0.689 | 0.788 |
| b_cells → cd68_macrophages | 0.4429 | 0.1150 | -0.3280 | 0.709 | 0.804 |
| plasma_cells → cd68_macrophages | 0.1222 | 0.1542 | 0.0320 | 0.728 | 0.819 |
| cd4_t_cells_cd45ro → vasculature | 1.3775 | 1.2201 | -0.1575 | 0.752 | 0.837 |

Continued on next page

| Cell type pair | CLR | DII | Effect size | p-value | adjusted p-value |
|---|---|---|---|---|---|
| stroma → tumor_cells | 2.4265 | 2.6656 | 0.2391 | 0.756 | 0.837 |
| stroma → cd68_cd163_macrophages | 4.3232 | 3.9593 | -0.3639 | 0.774 | 0.851 |
| stroma → stroma | 3.5335 | 3.2387 | -0.2947 | 0.784 | 0.856 |
| cd68_macrophages → cd8_t_cells | 0.2493 | 0.1942 | -0.0552 | 0.808 | 0.875 |
| smooth_muscle → cd68_macrophages | 0.0750 | 0.1041 | 0.0291 | 0.837 | 0.900 |
| tumor_cells → cd68_macrophages | 0.9647 | 1.0599 | 0.0953 | 0.852 | 0.909 |
| tumor_cells → stroma | 1.6059 | 1.4683 | -0.1376 | 0.861 | 0.909 |
| tumor_cells → smooth_muscle | -0.2772 | -0.1135 | 0.1637 | 0.865 | 0.909 |
| tregs → cd68_macrophages | 0.1272 | 0.1436 | 0.0164 | 0.874 | 0.912 |
| tumor_cells → cd4_t_cells_cd45ro | 0.2500 | 0.0627 | -0.1873 | 0.881 | 0.913 |
| cd68_macrophages → cd68_macrophages | 0.4182 | 0.3805 | -0.0377 | 0.889 | 0.914 |
| b_cells → stroma | 0.1103 | 0.2344 | 0.1240 | 0.904 | 0.924 |
| smooth_muscle → smooth_muscle | 4.9088 | 4.8654 | -0.0433 | 0.983 | 0.997 |
| granulocytes → b_cells | 1.5046 | 1.4927 | -0.0118 | 0.994 | 0.998 |
| tumor_cells → cd8_t_cells | 0.6589 | 0.6611 | 0.0022 | 0.998 | 0.998 |

## S8.8 CRC $p$-value distribution comparison

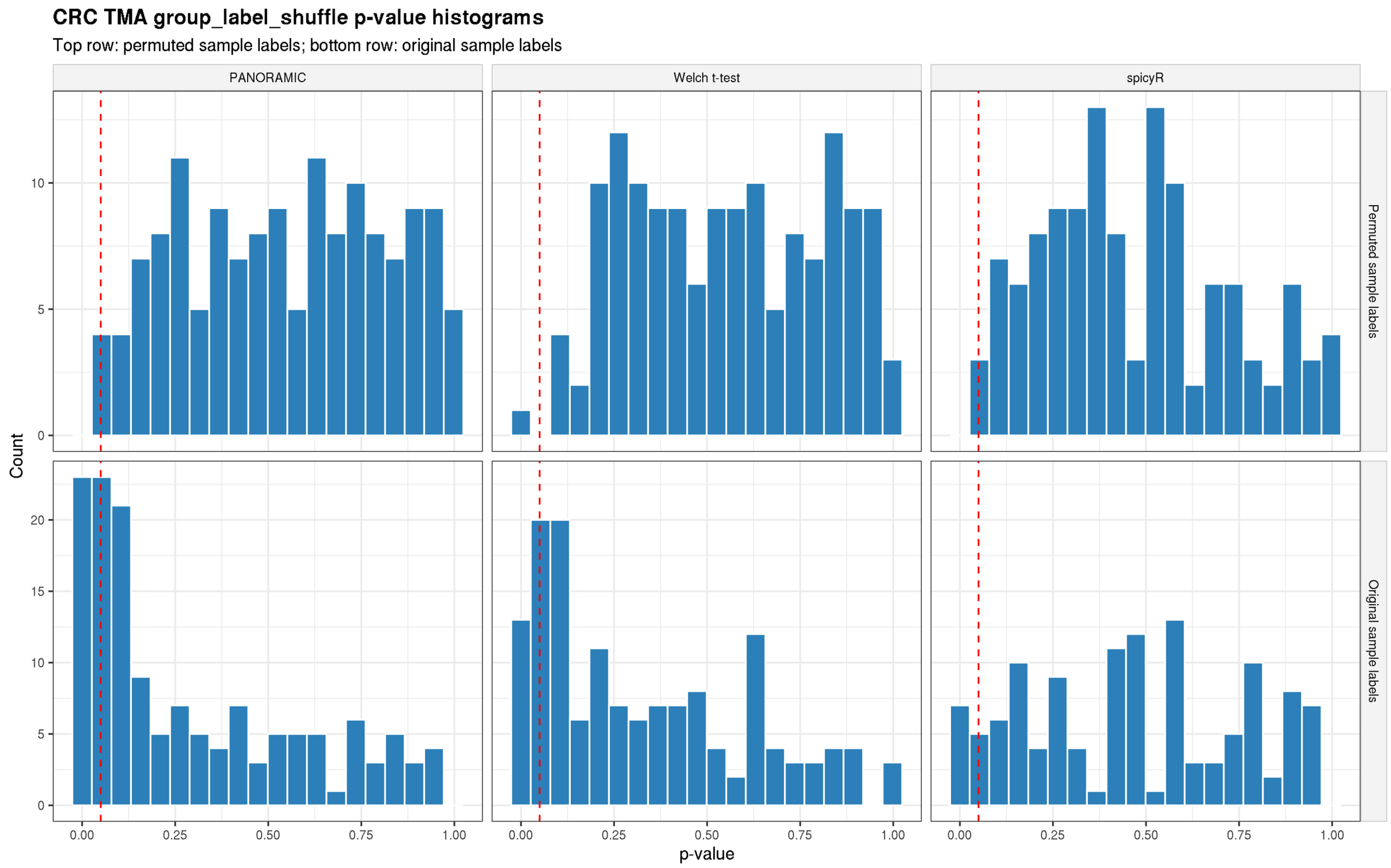


Figure S5: P-value distributions for CRC differential colocalization analyses at $r = 25\,\mu$m. Top row: p-value histograms of cell-type pair colocalization when sample labels (CLR or DII) are permuted. Bottom row: p-value histograms with correctly labeled data. PANORAMIC has improved sensitivity to colocalization differences over t-testing and spicyR.

## S8.9 Alternative spatial statistic: cross-$L$

Table S5: CRC TMA PANORAMIC results.

| Cell type pair | CLR | DII | Effect size | p-value | adjusted p-value |
|---|---|---|---|---|---|
| cd68_macrophages → cd68_macrophages | 82.2713 | 5.3709 | -76.9004 | 0.002 | 0.241 |
| smooth_muscle → smooth_muscle | 20.7612 | 4.9563 | -15.8048 | 0.077 | 0.999 |
| plasma_cells → cd68_macrophages | 35.5836 | 3.9282 | -31.6554 | 0.290 | 0.999 |
| cd68_cd163_macrophages → vasculature | 8.6311 | 0.5108 | -8.1202 | 0.326 | 0.999 |
| smooth_muscle → vasculature | 11.4141 | 1.2848 | -10.1293 | 0.382 | 0.999 |
| granulocytes → granulocytes | 10.4021 | 3.0199 | -7.3822 | 0.489 | 0.999 |
| vasculature → smooth_muscle | 11.1166 | 3.9819 | -7.1347 | 0.520 | 0.999 |
| granulocytes → vasculature | 8.1630 | 0.1581 | -8.0049 | 0.538 | 0.999 |
| plasma_cells → vasculature | 11.6652 | 2.0830 | -9.5822 | 0.574 | 0.999 |
| vasculature → granulocytes | 7.0033 | 0.6169 | -6.3864 | 0.596 | 0.999 |
| tumor_cells → vasculature | 2.9378 | -3.2650 | -6.2028 | 0.604 | 0.999 |
| cd8_t_cells → cd8_t_cells | 7.8677 | 1.9009 | -5.9667 | 0.607 | 0.999 |
| vasculature → vasculature | 19.1520 | 14.8440 | -4.3080 | 0.624 | 0.999 |
| plasma_cells → plasma_cells | 20.5571 | 12.1804 | -8.3767 | 0.631 | 0.999 |
| cd8_t_cells → vasculature | 6.4006 | -0.2359 | -6.6365 | 0.645 | 0.999 |
| cd68_cd163_macrophages → plasma_cells | 7.2653 | 2.1420 | -5.1233 | 0.647 | 0.999 |
| cd68_cd163_macrophages → tumor_cells | 2.6462 | -0.8011 | -3.4473 | 0.663 | 0.999 |
| tumor_cells → smooth_muscle | 3.6611 | -2.5311 | -6.1922 | 0.665 | 0.999 |
| plasma_cells → smooth_muscle | 10.0291 | 0.1604 | -9.8687 | 0.674 | 0.999 |
| cd8_t_cells → cd68_macrophages | 13.6388 | 1.8555 | -11.7833 | 0.674 | 0.999 |
| tumor_cells → cd68_cd163_macrophages | 1.4628 | -2.1447 | -3.6076 | 0.674 | 0.999 |
| tregs → stroma | 9.4794 | 2.2896 | -7.1898 | 0.685 | 0.999 |
| tumor_cells → stroma | 2.5686 | -1.2106 | -3.7792 | 0.689 | 0.999 |

Continued on next page

| Cell type pair | CLR | DII | Effect size | p-value | adjusted p-value |
|---|---|---|---|---|---|
| granulocytes → smooth_muscle | 5.1662 | -1.2240 | -6.3902 | 0.692 | 0.999 |
| tumor_cells → plasma_cells | 3.7170 | -1.9864 | -5.7034 | 0.693 | 0.999 |
| granulocytes → cd4_t_cells_cd45ro | 4.7072 | -0.1626 | -4.8698 | 0.694 | 0.999 |
| stroma → tregs | 9.0654 | 2.2130 | -6.8523 | 0.698 | 0.999 |
| cd4_t_cells_cd45ro → cd68_macrophages | 13.7313 | 3.2175 | -10.5138 | 0.701 | 0.999 |
| cd4_t_cells_cd45ro → b_cells | 4.1235 | 8.6041 | 4.4805 | 0.701 | 0.999 |
| smooth_muscle → granulocytes | 4.2396 | -0.7952 | -5.0348 | 0.716 | 0.999 |
| granulocytes → plasma_cells | 8.4914 | 2.5094 | -5.9819 | 0.717 | 0.999 |
| cd68_macrophages → plasma_cells | 17.5141 | 1.2552 | -16.2588 | 0.724 | 0.999 |
| stroma → smooth_muscle | 5.6620 | 2.5656 | -3.0964 | 0.725 | 0.999 |
| tumor_cells → tumor_cells | 2.4717 | 5.4653 | 2.9936 | 0.728 | 0.999 |
| cd68_cd163_macrophages → cd68_macrophages | 9.6964 | 2.8485 | -6.8479 | 0.729 | 0.999 |
| cd4_t_cells_cd45ro → smooth_muscle | 4.4706 | 0.0745 | -4.3961 | 0.729 | 0.999 |
| tumor_cells → cd8_t_cells | 1.3109 | -2.0916 | -3.4025 | 0.733 | 0.999 |
| cd68_cd163_macrophages → granulocytes | 3.7636 | 1.0677 | -2.6959 | 0.752 | 0.999 |
| vasculature → cd68_cd163_macrophages | 3.1168 | 0.3443 | -2.7725 | 0.766 | 0.999 |
| granulocytes → stroma | 3.9048 | 0.7077 | -3.1971 | 0.770 | 0.999 |
| vasculature → cd8_t_cells | 4.4522 | 1.0022 | -3.4500 | 0.771 | 0.999 |
| plasma_cells → tumor_cells | 3.2045 | -1.0390 | -4.2435 | 0.774 | 0.999 |
| tregs → b_cells | 5.1735 | 11.6221 | 6.4486 | 0.776 | 0.999 |
| granulocytes → cd8_t_cells | 3.5331 | 0.2876 | -3.2455 | 0.776 | 0.999 |
| cd8_t_cells → stroma | 3.9272 | 0.7687 | -3.1585 | 0.778 | 0.999 |
| granulocytes → tregs | 5.7231 | 0.6266 | -5.0965 | 0.781 | 0.999 |
| b_cells → plasma_cells | 0.5706 | 8.9437 | 8.3731 | 0.782 | 0.999 |
| tumor_cells → cd68_macrophages | 7.8693 | 2.3931 | -5.4762 | 0.784 | 0.999 |
| cd68_macrophages → cd68_cd163_macrophages | 7.1938 | 1.6413 | -5.5525 | 0.786 | 0.999 |

Continued on next page

| Cell type pair | CLR | DII | Effect size | p-value | adjusted p-value |
|---|---|---|---|---|---|
| stroma → granulocytes | 3.0050 | 0.4379 | -2.5671 | 0.786 | 0.999 |
| smooth_muscle → stroma | 4.0816 | 1.1698 | -2.9118 | 0.793 | 0.999 |
| tumor_cells → b_cells | 2.9429 | -0.6576 | -3.6005 | 0.798 | 0.999 |
| cd8_t_cells → cd4_t_cells_cd45ro | 6.6073 | 3.8132 | -2.7940 | 0.801 | 0.999 |
| vasculature → tumor_cells | 1.3609 | -1.8534 | -3.2143 | 0.805 | 0.999 |
| granulocytes → cd68_macrophages | 13.3201 | 7.6703 | -5.6498 | 0.806 | 0.999 |
| tumor_cells → cd4_t_cells_cd45ro | 1.5601 | -0.8209 | -2.3809 | 0.807 | 0.999 |
| smooth_muscle → cd68_macrophages | -3.5159 | 1.5897 | 5.1056 | 0.822 | 0.999 |
| granulocytes → b_cells | 4.0621 | 0.7280 | -3.3341 | 0.828 | 0.999 |
| cd68_cd163_macrophages → b_cells | 3.0134 | 5.2233 | 2.2100 | 0.829 | 0.999 |
| cd68_macrophages → cd4_t_cells_cd45ro | 7.8428 | 2.0642 | -5.7786 | 0.833 | 0.999 |
| cd4_t_cells_cd45ro → vasculature | 4.2365 | 1.8185 | -2.4179 | 0.833 | 0.999 |
| b_cells → b_cells | 10.5905 | 6.3983 | -4.1922 | 0.836 | 0.999 |
| b_cells → granulocytes | 4.6641 | -0.2091 | -4.8732 | 0.837 | 0.999 |
| smooth_muscle → plasma_cells | 4.7296 | -0.3190 | -5.0486 | 0.838 | 0.999 |
| cd8_t_cells → tumor_cells | 1.5979 | -0.7588 | -2.3567 | 0.843 | 0.999 |
| stroma → cd4_t_cells_cd45ro | 1.0535 | 2.5860 | 1.5325 | 0.852 | 0.999 |
| plasma_cells → cd68_cd163_macrophages | 4.2469 | 1.8409 | -2.4061 | 0.856 | 0.999 |
| b_cells → cd68_cd163_macrophages | -1.6254 | 1.8632 | 3.4886 | 0.856 | 0.999 |
| plasma_cells → granulocytes | 4.2460 | 1.1336 | -3.1124 | 0.858 | 0.999 |
| cd68_macrophages → granulocytes | 10.7467 | 6.7849 | -3.9618 | 0.858 | 0.999 |
| cd68_macrophages → stroma | 6.4970 | 2.7474 | -3.7496 | 0.864 | 0.999 |
| granulocytes → cd68_cd163_macrophages | 1.5601 | -0.1294 | -1.6895 | 0.864 | 0.999 |
| cd68_macrophages → cd8_t_cells | 4.7308 | 0.0822 | -4.6486 | 0.865 | 0.999 |
| smooth_muscle → cd8_t_cells | -1.0554 | 1.2928 | 2.3483 | 0.869 | 0.999 |
| stroma → plasma_cells | 1.7795 | 4.1235 | 2.3440 | 0.874 | 0.999 |

Continued on next page

| Cell type pair | CLR | DII | Effect size | p-value | adjusted p-value |
|---|---|---|---|---|---|
| b_cells → vasculature | 0.8027 | 4.1912 | 3.3885 | 0.874 | 0.999 |
| tumor_cells → tregs | 2.0340 | -0.6694 | -2.7035 | 0.874 | 0.999 |
| smooth_muscle → b_cells | -3.8283 | -0.5784 | 3.2499 | 0.876 | 0.999 |
| cd8_t_cells → cd68_cd163_macrophages | 3.6131 | 2.1121 | -1.5010 | 0.878 | 0.999 |
| vasculature → plasma_cells | 6.5907 | 4.1125 | -2.4782 | 0.890 | 0.999 |
| cd68_cd163_macrophages → smooth_muscle | 2.8082 | 1.3949 | -1.4133 | 0.891 | 0.999 |
| stroma → cd68_cd163_macrophages | 1.6576 | 0.7572 | -0.9004 | 0.893 | 0.999 |
| plasma_cells → tregs | 5.3226 | 10.5450 | 5.2225 | 0.893 | 0.999 |
| smooth_muscle → tregs | -3.1744 | 1.2604 | 4.4348 | 0.896 | 0.999 |
| b_cells → cd68_macrophages | 24.2108 | 17.6836 | -6.5272 | 0.897 | 0.999 |
| plasma_cells → cd8_t_cells | 5.0542 | 3.0702 | -1.9840 | 0.897 | 0.999 |
| cd8_t_cells → b_cells | 8.7394 | 6.9260 | -1.8134 | 0.898 | 0.999 |
| cd4_t_cells_cd45ro → cd8_t_cells | 4.1312 | 3.0088 | -1.1224 | 0.902 | 0.999 |
| tregs → plasma_cells | 12.0239 | 7.6338 | -4.3901 | 0.906 | 0.999 |
| stroma → tumor_cells | 2.4921 | 1.5168 | -0.9753 | 0.909 | 0.999 |
| stroma → b_cells | 0.8437 | 2.1954 | 1.3517 | 0.912 | 0.999 |
| plasma_cells → cd4_t_cells_cd45ro | 6.0266 | 4.5041 | -1.5225 | 0.916 | 0.999 |
| cd8_t_cells → plasma_cells | 6.1128 | 4.3909 | -1.7218 | 0.917 | 0.999 |
| cd8_t_cells → granulocytes | 3.1106 | 1.5849 | -1.5257 | 0.918 | 0.999 |
| cd68_macrophages → smooth_muscle | 2.0890 | 5.1769 | 3.0879 | 0.919 | 0.999 |
| vasculature → cd4_t_cells_cd45ro | 3.6903 | 2.5713 | -1.1189 | 0.919 | 0.999 |
| plasma_cells → stroma | 1.3750 | 2.8778 | 1.5028 | 0.919 | 0.999 |
| vasculature → tregs | -0.0623 | 2.8527 | 2.9150 | 0.921 | 0.999 |
| tregs → tregs | 11.1620 | 7.6627 | -3.4993 | 0.925 | 0.999 |
| b_cells → tumor_cells | 2.8742 | 0.9338 | -1.9403 | 0.927 | 0.999 |
| b_cells → smooth_muscle | -2.5461 | 0.3992 | 2.9453 | 0.930 | 0.999 |
| cd8_t_cells → tregs | 6.9962 | 5.2921 | -1.7040 | 0.932 | 0.999 |
| cd68_cd163_macrophages → tregs | 2.7315 | 4.4656 | 1.7341 | 0.932 | 0.999 |
| stroma → stroma | -0.1426 | 0.4478 | 0.5903 | 0.935 | 0.999 |
| vasculature → b_cells | 1.5698 | 2.8203 | 1.2505 | 0.939 | 0.999 |

Continued on next page

| Cell type pair | CLR | DII | Effect size | p-value | adjusted p-value |
|---|---|---|---|---|---|
| tregs → cd68_cd163_macrophages | 4.4954 | 3.2653 | -1.2301 | 0.940 | 0.999 |
| cd4_t_cells_cd45ro → cd4_t_cells_cd45ro | 1.9091 | 1.2753 | -0.6338 | 0.942 | 0.999 |
| smooth_muscle → tumor_cells | 0.3756 | -0.6882 | -1.0638 | 0.942 | 0.999 |
| b_cells → cd8_t_cells | 7.1118 | 5.8068 | -1.3050 | 0.945 | 0.999 |
| cd4_t_cells_cd45ro → tregs | 3.2353 | 4.3175 | 1.0822 | 0.946 | 0.999 |
| cd68_macrophages → tumor_cells | 2.8725 | 1.3930 | -1.4796 | 0.949 | 0.999 |
| tregs → smooth_muscle | 3.3038 | 1.8928 | -1.4110 | 0.951 | 0.999 |
| tumor_cells → granulocytes | 0.5013 | -0.1050 | -0.6063 | 0.954 | 0.999 |
| plasma_cells → b_cells | 7.8005 | 8.9311 | 1.1306 | 0.954 | 0.999 |
| cd8_t_cells → smooth_muscle | 0.6374 | 1.5413 | 0.9039 | 0.955 | 0.999 |
| smooth_muscle → cd4_t_cells_cd45ro | 1.0649 | 0.3778 | -0.6871 | 0.956 | 0.999 |
| granulocytes → tumor_cells | 0.6889 | 0.0128 | -0.6761 | 0.956 | 0.999 |
| cd4_t_cells_cd45ro → cd68_cd163_macrophages | 1.0122 | 1.4760 | 0.4638 | 0.958 | 0.999 |
| tregs → cd68_macrophages | 3.0549 | 4.7286 | 1.6737 | 0.958 | 0.999 |
| tregs → cd8_t_cells | 5.4228 | 4.4752 | -0.9476 | 0.959 | 0.999 |
| cd68_macrophages → b_cells | 7.3006 | 9.3806 | 2.0800 | 0.959 | 0.999 |
| b_cells → tregs | 10.7686 | 9.1757 | -1.5929 | 0.960 | 0.999 |
| cd68_cd163_macrophages → stroma | 0.8192 | 0.4637 | -0.3555 | 0.962 | 0.999 |
| stroma → cd68_macrophages | 3.7232 | 2.9317 | -0.7916 | 0.963 | 0.999 |
| smooth_muscle → cd68_cd163_macrophages | 0.2394 | 0.7406 | 0.5012 | 0.964 | 0.999 |
| cd68_cd163_macrophages → cd8_t_cells | 2.5839 | 2.9419 | 0.3580 | 0.964 | 0.999 |
| tregs → granulocytes | 2.2992 | 1.3029 | -0.9963 | 0.964 | 0.999 |
| cd4_t_cells_cd45ro → tumor_cells | 0.1942 | -0.2614 | -0.4556 | 0.965 | 0.999 |
| b_cells → stroma | 1.4748 | 0.6694 | -0.8054 | 0.967 | 0.999 |
| stroma → vasculature | 2.9399 | 2.5780 | -0.3618 | 0.969 | 0.999 |
| tregs → cd4_t_cells_cd45ro | 4.9632 | 4.2671 | -0.6961 | 0.970 | 0.999 |
| vasculature → cd68_macrophages | -0.1546 | 0.8791 | 1.0336 | 0.971 | 0.999 |
| tregs → vasculature | 1.9642 | 2.8611 | 0.8970 | 0.973 | 0.999 |

Continued on next page

| Cell type pair | CLR | DII | Effect size | p-value | adjusted p-value |
|---|---|---|---|---|---|
| cd4_t_cells_cd45ro → granulocytes | 0.8999 | 1.2571 | 0.3571 | 0.974 | 0.999 |
| b_cells → cd4_t_cells_cd45ro | 6.5331 | 7.0917 | 0.5586 | 0.975 | 0.999 |
| cd68_cd163_macrophages → cd4_t_cells_cd45ro | 1.7435 | 1.9904 | 0.2469 | 0.976 | 0.999 |
| cd68_cd163_macrophages → cd68_cd163_macrophages | 1.1211 | 1.3064 | 0.1853 | 0.977 | 0.999 |
| cd4_t_cells_cd45ro → plasma_cells | 5.0204 | 5.3836 | 0.3632 | 0.982 | 0.999 |
| cd4_t_cells_cd45ro → stroma | 2.5637 | 2.3829 | -0.1808 | 0.986 | 0.999 |
| vasculature → stroma | 2.8007 | 2.6361 | -0.1646 | 0.988 | 0.999 |
| cd68_macrophages → tregs | 1.6504 | 1.1485 | -0.5019 | 0.992 | 0.999 |
| cd68_macrophages → vasculature | 3.2834 | 3.1395 | -0.1438 | 0.997 | 0.999 |
| tregs → tumor_cells | 0.8129 | 0.7451 | -0.0678 | 0.997 | 0.999 |
| stroma → cd8_t_cells | 1.2013 | 1.2167 | 0.0154 | 0.999 | 0.999 |

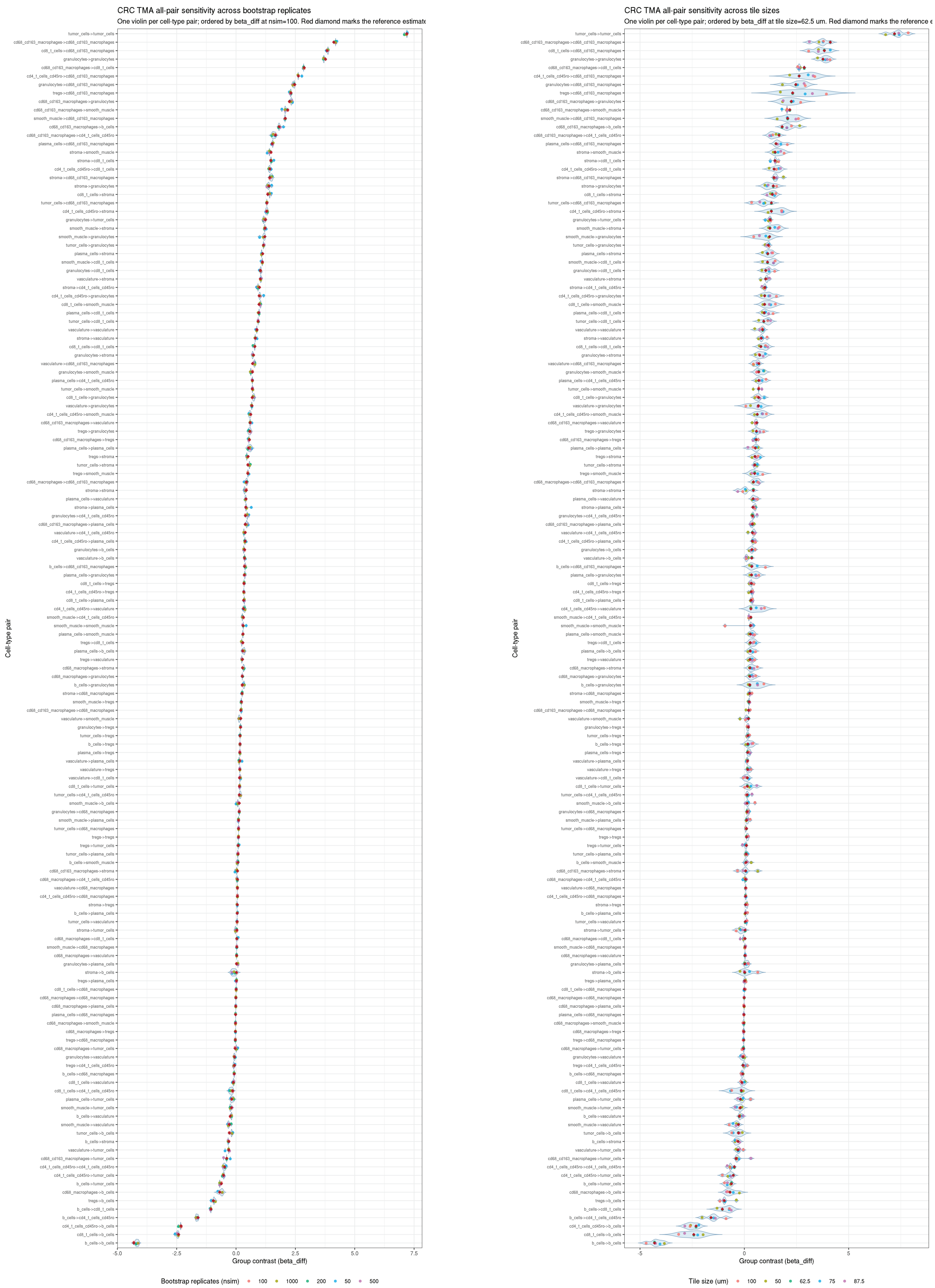


Figure S6: Left: Sensitivity analysis for the number of bootstrap iterations. Right: Sensitivity analysis for the choice of tile size in the spatial bootstrap.

### S8.10 Sensitivity analysis

## S9 HNSCC supplementary results

### S9.1 HNSCC cohort overview

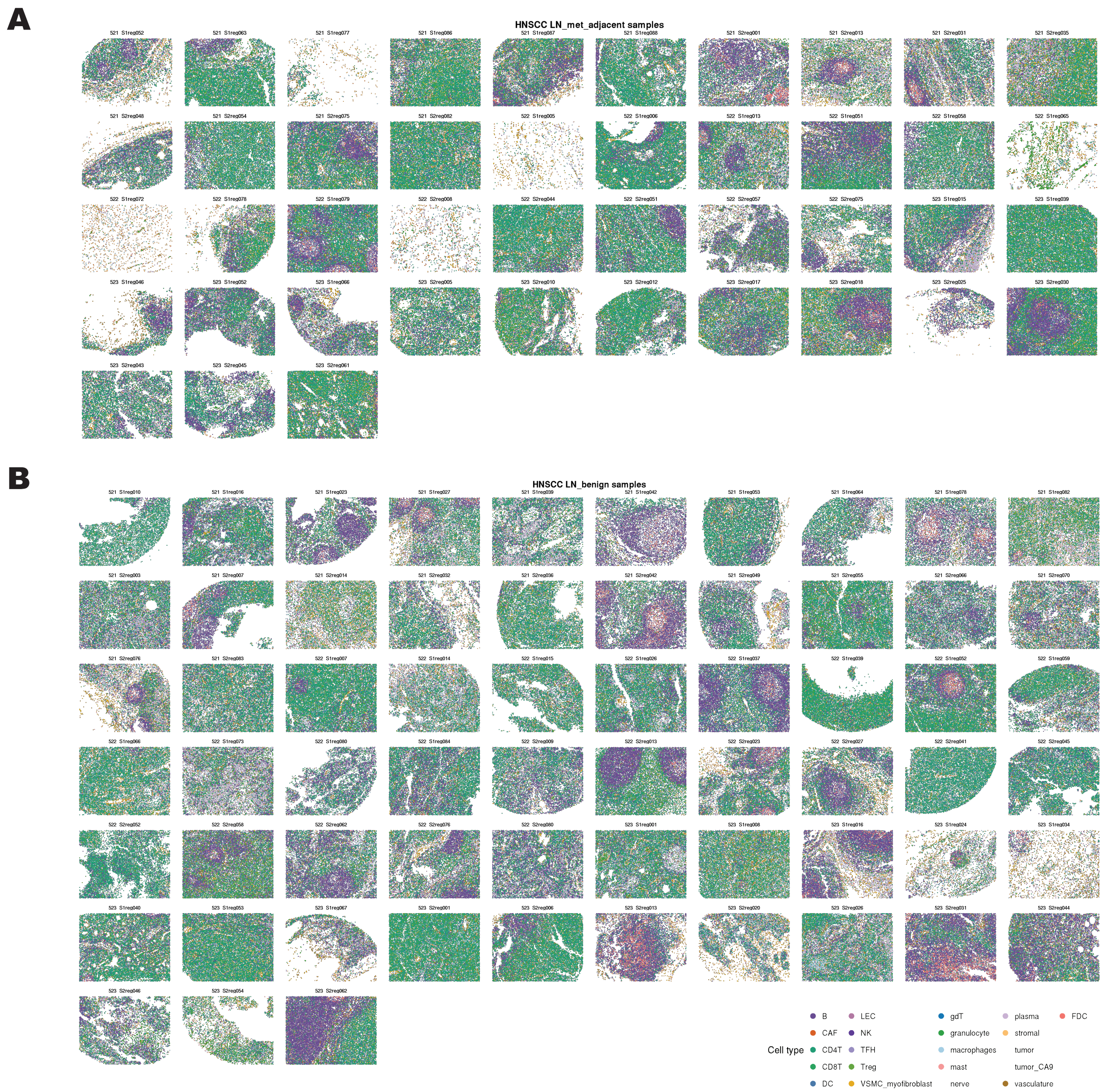


Figure S7: Overview of HNSCC lymph node TMA samples used in the PANORAMIC analysis. Each panel shows spatial cell maps from the Haist et al. HNSCC cohort, with points representing single cells colored by annotated cell type. (A) Tumor-adjacent lymph node regions from patients with lymph node metastasis. (B) Benign lymph node samples from node-negative patients.

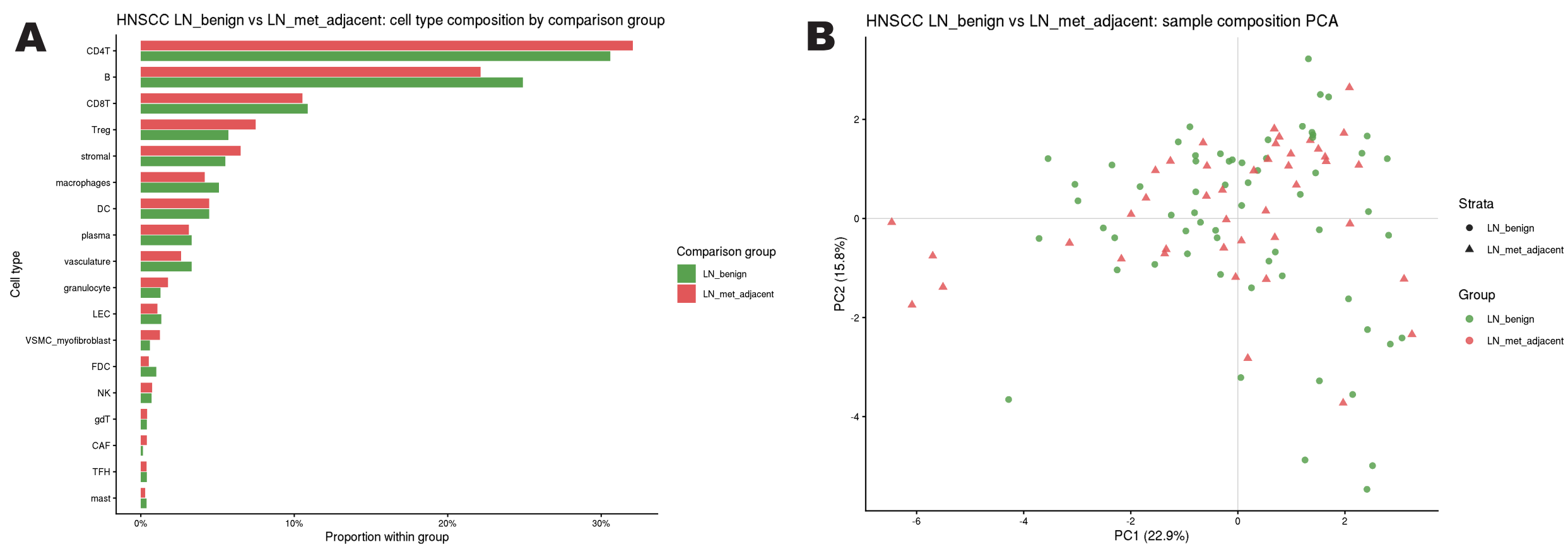


Figure S8: Overview of HNSCC lymph node cell-type proportions. (A) Proportions of retained cell types between benign lymph node and tumor-adjacent lymph node regions. (B) PC1 and PC2 generated from the matrix of cell-type proportions per sampled region.

## S9.2 Full PANORAMIC HNSCC results at 25 $\mu$m

Table S6: HNSCC TMA PANORAMIC results, sorted by p-value.

| Cell type pair | LN benign | LN met-adjacent | Effect size | p-value | adjusted p-value |
|---|---|---|---|---|---|
| NK → gdT | -0.0052 | 0.0356 | 0.0408 | 0.000 | 0.000 |
| mast → CD8T | 0.2075 | 0.0710 | -0.1365 | 0.000 | 0.056 |
| LEC → mast | 0.0508 | -0.0078 | -0.0586 | 0.001 | 0.102 |
| mast → B | 0.1934 | 0.0820 | -0.1115 | 0.007 | 0.369 |
| FDC → vasculature | -0.3090 | -0.0593 | 0.2497 | 0.007 | 0.369 |
| vasculature → LEC | 0.8627 | 0.4239 | -0.4387 | 0.009 | 0.369 |
| gdT → DC | 0.0461 | 0.1042 | 0.0581 | 0.010 | 0.369 |
| DC → granulocyte | 0.6154 | 0.2615 | -0.3539 | 0.011 | 0.369 |
| plasma → CD4T | 1.3842 | 0.4078 | -0.9765 | 0.011 | 0.369 |
| mast → LEC | 0.0508 | 0.0011 | -0.0497 | 0.013 | 0.369 |
| granulocyte → DC | 0.4371 | 0.1987 | -0.2384 | 0.014 | 0.369 |
| B → mast | 1.3935 | 0.5647 | -0.8288 | 0.015 | 0.369 |
| FDC → FDC | 1.0216 | 0.4240 | -0.5976 | 0.016 | 0.369 |
| vasculature → B | 1.3658 | 0.9281 | -0.4377 | 0.017 | 0.369 |
| mast → stromal | 0.1516 | 0.0633 | -0.0884 | 0.019 | 0.369 |
| stromal → gdT | 0.2872 | 0.5936 | 0.3064 | 0.019 | 0.369 |
| CD8T → mast | 0.7887 | 0.3797 | -0.4090 | 0.020 | 0.369 |
| TFH → LEC | -0.0890 | -0.0301 | 0.0589 | 0.023 | 0.369 |
| plasma → B | 1.1945 | 0.4894 | -0.7051 | 0.024 | 0.369 |
| plasma → Treg | 1.1132 | 0.4511 | -0.6621 | 0.024 | 0.369 |
| vasculature → TFH | -0.0881 | 0.0036 | 0.0916 | 0.027 | 0.395 |
| FDC → TFH | 0.3412 | 0.1179 | -0.2233 | 0.032 | 0.395 |
| mast → Treg | 0.1407 | 0.0591 | -0.0816 | 0.033 | 0.395 |
| granulocyte → VSMC_myofibroblast | -0.0158 | -0.0078 | 0.0080 | 0.033 | 0.395 |
| mast → granulocyte | 0.0371 | 0.0003 | -0.0368 | 0.034 | 0.395 |
| TFH → TFH | 0.3541 | 0.1352 | -0.2189 | 0.034 | 0.395 |
| FDC → DC | 0.1280 | 0.0119 | -0.1160 | 0.035 | 0.395 |

Continued on next page

| Cell type pair | LN benign | LN met-adjacent | Effect size | p-value | adjusted p-value |
|---|---|---|---|---|---|
| plasma → macrophages | 1.4258 | 0.5568 | -0.8690 | 0.036 | 0.395 |
| VSMC_myofibroblast → TFH | -0.0288 | -0.0120 | 0.0168 | 0.039 | 0.395 |
| CD8T → DC | 4.4046 | 3.1154 | -1.2892 | 0.039 | 0.395 |
| macrophages → mast | 0.7480 | 0.3907 | -0.3573 | 0.041 | 0.395 |
| vasculature → gdT | 0.1392 | 0.2630 | 0.1238 | 0.044 | 0.395 |
| granulocyte → mast | 0.0473 | -0.0028 | -0.0500 | 0.046 | 0.395 |
| TFH → FDC | 0.6042 | 0.2360 | -0.3682 | 0.046 | 0.395 |
| mast → FDC | -0.0153 | -0.0084 | 0.0069 | 0.046 | 0.395 |
| granulocyte → Treg | 0.3259 | 0.1624 | -0.1635 | 0.048 | 0.395 |
| CAF → Treg | 0.0907 | 0.0436 | -0.0470 | 0.048 | 0.395 |
| CD4T → mast | 1.6960 | 0.8428 | -0.8532 | 0.050 | 0.395 |
| FDC → B | 0.1634 | 0.0665 | -0.0969 | 0.051 | 0.395 |
| VSMC_myofibroblast → CAF | -0.0286 | -0.0058 | 0.0228 | 0.052 | 0.395 |
| gdT → CD8T | 0.1016 | 0.1794 | 0.0779 | 0.052 | 0.395 |
| CAF → FDC | -0.0135 | -0.0061 | 0.0075 | 0.054 | 0.404 |
| mast → CD4T | 0.1496 | 0.0845 | -0.0651 | 0.056 | 0.407 |
| vasculature → granulocyte | 1.1833 | 0.7593 | -0.4241 | 0.060 | 0.422 |
| FDC → stromal | -0.0786 | -0.0070 | 0.0717 | 0.061 | 0.422 |
| vasculature → plasma | 0.9223 | 0.6055 | -0.3168 | 0.064 | 0.427 |
| CAF → macrophages | 0.1020 | 0.0448 | -0.0572 | 0.064 | 0.427 |
| gdT → NK | 0.0044 | 0.0295 | 0.0251 | 0.071 | 0.459 |
| vasculature → CD4T | 1.4172 | 1.0550 | -0.3622 | 0.074 | 0.461 |
| gdT → macrophages | 0.0687 | 0.1265 | 0.0578 | 0.076 | 0.461 |
| LEC → vasculature | 0.5658 | 0.3097 | -0.2562 | 0.078 | 0.461 |
| CAF → DC | 0.0743 | 0.0296 | -0.0447 | 0.078 | 0.461 |
| TFH → vasculature | -0.0840 | -0.0106 | 0.0733 | 0.078 | 0.461 |
| TFH → granulocyte | -0.0659 | -0.0230 | 0.0429 | 0.080 | 0.463 |
| gdT → gdT | 0.0253 | 0.0666 | 0.0413 | 0.087 | 0.463 |
| vasculature → DC | 0.9418 | 0.6589 | -0.2829 | 0.087 | 0.463 |
| B → FDC | 7.2431 | 4.4550 | -2.7881 | 0.088 | 0.463 |
| stromal → mast | 0.6527 | 0.3683 | -0.2844 | 0.089 | 0.463 |
| plasma → VSMC_myofibroblast | 0.0844 | 0.2144 | 0.1300 | 0.090 | 0.463 |
| CAF → gdT | -0.0131 | -0.0051 | 0.0080 | 0.090 | 0.463 |
| plasma → plasma | 3.2888 | 1.9636 | -1.3252 | 0.092 | 0.463 |
| LEC → TFH | -0.0457 | -0.0221 | 0.0236 | 0.092 | 0.463 |
| CD4T → TFH | 0.5317 | 1.1378 | 0.6061 | 0.094 | 0.464 |
| TFH → CAF | -0.0252 | -0.0024 | 0.0227 | 0.099 | 0.475 |
| gdT → stromal | 0.0693 | 0.1198 | 0.0505 | 0.099 | 0.475 |
| TFH → VSMC_myofibroblast | -0.0563 | -0.0143 | 0.0420 | 0.101 | 0.475 |
| Treg → CAF | 0.3162 | 0.1832 | -0.1330 | 0.102 | 0.475 |
| mast → TFH | -0.0108 | -0.0056 | 0.0052 | 0.106 | 0.475 |

Continued on next page

| Cell type pair | LN benign | LN met-adjacent | Effect size | p-value | adjusted p-value |
|---|---|---|---|---|---|
| CAF → CD8T | 0.0859 | 0.0512 | -0.0346 | 0.106 | 0.475 |
| FDC → CAF | -0.0642 | -0.0252 | 0.0390 | 0.107 | 0.475 |
| DC → vasculature | 1.1080 | 0.6809 | -0.4271 | 0.110 | 0.475 |
| FDC → mast | -0.0440 | -0.0209 | 0.0231 | 0.110 | 0.475 |
| CAF → CAF | -0.0099 | -0.0023 | 0.0076 | 0.114 | 0.480 |
| CD8T → CD8T | 4.8470 | 3.6991 | -1.1479 | 0.114 | 0.480 |
| Treg → NK | 0.7184 | 1.1035 | 0.3851 | 0.115 | 0.480 |
| TFH → DC | 0.0186 | 0.0534 | 0.0348 | 0.118 | 0.486 |
| gdT → vasculature | 0.0472 | 0.0900 | 0.0428 | 0.123 | 0.499 |
| DC → macrophages | 1.8226 | 1.3569 | -0.4657 | 0.134 | 0.520 |
| mast → CAF | 0.0275 | -0.0069 | -0.0344 | 0.136 | 0.520 |
| NK → NK | 0.0682 | 0.1268 | 0.0586 | 0.140 | 0.520 |
| plasma → stromal | 1.0402 | 0.5513 | -0.4889 | 0.140 | 0.520 |
| granulocyte → plasma | 0.1773 | 0.0675 | -0.1097 | 0.140 | 0.520 |
| TFH → mast | -0.0065 | -0.0043 | 0.0022 | 0.141 | 0.520 |
| mast → DC | 0.0652 | 0.0256 | -0.0396 | 0.141 | 0.520 |
| granulocyte → NK | 0.0585 | 0.0139 | -0.0446 | 0.142 | 0.520 |
| CD4T → NK | 3.7663 | 5.0825 | 1.3162 | 0.145 | 0.525 |
| gdT → CAF | -0.0077 | -0.0050 | 0.0027 | 0.150 | 0.539 |
| plasma → FDC | 0.0106 | -0.0433 | -0.0539 | 0.155 | 0.544 |
| NK → plasma | 0.0994 | 0.1823 | 0.0830 | 0.156 | 0.544 |
| macrophages → VSMC_myofibroblast | 0.1429 | 0.4870 | 0.3441 | 0.159 | 0.544 |
| gdT → LEC | 0.0243 | 0.0033 | -0.0210 | 0.159 | 0.544 |
| CD4T → gdT | 2.2393 | 3.2978 | 1.0585 | 0.163 | 0.551 |
| CAF → vasculature | 0.1176 | 0.0708 | -0.0468 | 0.169 | 0.563 |
| vasculature → macrophages | 1.4556 | 1.1238 | -0.3318 | 0.172 | 0.563 |
| TFH → macrophages | -0.0700 | -0.0073 | 0.0627 | 0.172 | 0.563 |
| TFH → Treg | 0.0191 | 0.0518 | 0.0327 | 0.173 | 0.563 |
| granulocyte → gdT | -0.0042 | -0.0078 | -0.0036 | 0.176 | 0.563 |
| FDC → macrophages | -0.2067 | -0.0778 | 0.1290 | 0.177 | 0.563 |
| vasculature → FDC | -0.0822 | -0.0291 | 0.0532 | 0.179 | 0.563 |
| VSMC_myofibroblast → LEC | -0.0080 | -0.0132 | -0.0052 | 0.185 | 0.574 |
| CD8T → macrophages | 4.1853 | 3.2709 | -0.9144 | 0.186 | 0.574 |
| mast → macrophages | 0.2050 | 0.1339 | -0.0711 | 0.193 | 0.592 |
| FDC → granulocyte | -0.1509 | -0.0541 | 0.0968 | 0.195 | 0.592 |
| CD8T → stromal | 4.5700 | 3.5891 | -0.9809 | 0.200 | 0.601 |
| macrophages → FDC | 0.0155 | -0.0363 | -0.0518 | 0.202 | 0.602 |
| macrophages → DC | 1.7957 | 1.4327 | -0.3630 | 0.207 | 0.608 |
| gdT → B | 0.1043 | 0.1478 | 0.0434 | 0.208 | 0.608 |
| NK → VSMC_myofibroblast | -0.0077 | 0.0104 | 0.0181 | 0.218 | 0.625 |
| DC → mast | 0.1531 | 0.0782 | -0.0749 | 0.221 | 0.625 |

Continued on next page

| Cell type pair | LN benign | LN met-adjacent | Effect size | p-value | adjusted p-value |
|---|---|---|---|---|---|
| NK → granulocyte | 0.0711 | 0.0394 | -0.0317 | 0.221 | 0.625 |
| Treg → VSMC_myofibroblast | 0.1689 | 0.5195 | 0.3506 | 0.222 | 0.625 |
| granulocyte → TFH | -0.0581 | -0.0203 | 0.0378 | 0.226 | 0.627 |
| DC → DC | 2.3698 | 1.8689 | -0.5009 | 0.227 | 0.627 |
| vasculature → CAF | 0.2705 | 0.1930 | -0.0774 | 0.231 | 0.633 |
| CD8T → CD4T | 5.5276 | 4.5914 | -0.9362 | 0.236 | 0.640 |
| DC → CD8T | 2.0241 | 1.5619 | -0.4622 | 0.240 | 0.642 |
| granulocyte → FDC | -0.0462 | -0.0189 | 0.0272 | 0.242 | 0.642 |
| DC → stromal | 2.0353 | 1.5254 | -0.5099 | 0.243 | 0.642 |
| plasma → vasculature | 1.0393 | 0.6915 | -0.3477 | 0.245 | 0.642 |
| vasculature → stromal | 1.8733 | 1.6131 | -0.2602 | 0.247 | 0.642 |
| gdT → plasma | 0.0553 | 0.0851 | 0.0298 | 0.251 | 0.648 |
| stromal → TFH | 0.0978 | 0.1454 | 0.0476 | 0.257 | 0.651 |
| CD8T → Treg | 4.9554 | 4.0295 | -0.9259 | 0.258 | 0.651 |
| B → B | 11.8686 | 9.5080 | -2.3607 | 0.259 | 0.651 |
| stromal → CAF | 0.5579 | 0.9405 | 0.3826 | 0.261 | 0.652 |
| plasma → LEC | -0.0500 | 0.0408 | 0.0909 | 0.269 | 0.666 |
| gdT → FDC | -0.0082 | -0.0121 | -0.0040 | 0.277 | 0.681 |
| gdT → TFH | -0.0058 | -0.0108 | -0.0050 | 0.285 | 0.694 |
| vasculature → mast | 0.1899 | 0.1252 | -0.0647 | 0.291 | 0.702 |
| DC → gdT | 0.2523 | 0.3340 | 0.0817 | 0.296 | 0.702 |
| CD8T → CAF | 0.5728 | 0.4088 | -0.1640 | 0.302 | 0.702 |
| vasculature → CD8T | 1.3382 | 1.1103 | -0.2280 | 0.302 | 0.702 |
| CD4T → VSMC_myofibroblast | 1.1853 | 2.6596 | 1.4743 | 0.308 | 0.702 |
| Treg → TFH | 0.2597 | 0.3965 | 0.1369 | 0.309 | 0.702 |
| plasma → NK | 0.1107 | 0.1825 | 0.0718 | 0.309 | 0.702 |
| VSMC_myofibroblast → macrophages | 0.1777 | 0.4251 | 0.2474 | 0.309 | 0.702 |
| mast → plasma | 0.0655 | 0.0382 | -0.0273 | 0.310 | 0.702 |
| VSMC_myofibroblast → Treg | 0.1456 | 0.3890 | 0.2434 | 0.311 | 0.702 |
| NK → CD8T | 0.2469 | 0.3155 | 0.0686 | 0.313 | 0.702 |
| CD8T → vasculature | 2.8328 | 2.3013 | -0.5316 | 0.320 | 0.713 |
| plasma → mast | -0.0086 | 0.0075 | 0.0161 | 0.327 | 0.716 |
| macrophages → vasculature | 1.8684 | 1.4704 | -0.3980 | 0.330 | 0.716 |
| CAF → TFH | -0.0077 | -0.0055 | 0.0021 | 0.331 | 0.716 |
| CD8T → gdT | 1.1923 | 1.6453 | 0.4530 | 0.331 | 0.716 |
| DC → CD4T | 2.2127 | 1.8332 | -0.3795 | 0.335 | 0.716 |
| B → TFH | 5.1196 | 3.6144 | -1.5052 | 0.337 | 0.716 |
| B → DC | 8.9801 | 7.3432 | -1.6369 | 0.341 | 0.716 |
| CD4T → granulocyte | 2.2006 | 1.4628 | -0.7377 | 0.342 | 0.716 |
| gdT → CD4T | 0.1352 | 0.1739 | 0.0387 | 0.345 | 0.716 |
| Treg → granulocyte | 0.3260 | 0.2124 | -0.1136 | 0.347 | 0.716 |

Continued on next page

| Cell type pair | LN benign | LN met-adjacent | Effect size | p-value | adjusted p-value |
|---|---|---|---|---|---|
| CD4T → Treg | 10.1896 | 12.5242 | 2.3346 | 0.348 | 0.716 |
| VSMC_myofibroblast → vasculature | 0.4000 | 0.6032 | 0.2032 | 0.349 | 0.716 |
| Treg → gdT | 0.5537 | 0.6892 | 0.1355 | 0.351 | 0.716 |
| macrophages → LEC | 1.4595 | 1.0913 | -0.3682 | 0.360 | 0.721 |
| CAF → stromal | 0.1032 | 0.0731 | -0.0301 | 0.361 | 0.721 |
| VSMC_myofibroblast → FDC | -0.0213 | -0.0123 | 0.0090 | 0.362 | 0.721 |
| NK → Treg | 0.2142 | 0.2742 | 0.0599 | 0.366 | 0.721 |
| macrophages → gdT | 0.3626 | 0.4747 | 0.1122 | 0.369 | 0.721 |
| B → VSMC_myofibroblast | -0.3251 | 0.2047 | 0.5298 | 0.369 | 0.721 |
| VSMC_myofibroblast → VSMC_myofibroblast | 0.7175 | 1.0544 | 0.3369 | 0.371 | 0.721 |
| CD4T → B | 9.6399 | 11.3050 | 1.6651 | 0.372 | 0.721 |
| CD8T → B | 4.7519 | 4.0676 | -0.6843 | 0.376 | 0.725 |
| DC → CAF | 0.1447 | 0.0835 | -0.0612 | 0.381 | 0.729 |
| CAF → mast | 0.0085 | -0.0039 | -0.0124 | 0.385 | 0.733 |
| FDC → LEC | -0.1168 | -0.0380 | 0.0788 | 0.390 | 0.733 |
| VSMC_myofibroblast → CD4T | 0.2455 | 0.4751 | 0.2297 | 0.390 | 0.733 |
| DC → LEC | 0.5825 | 0.4608 | -0.1217 | 0.397 | 0.742 |
| VSMC_myofibroblast → DC | 0.1279 | 0.2899 | 0.1619 | 0.402 | 0.746 |
| CAF → CD4T | 0.0720 | 0.0552 | -0.0168 | 0.407 | 0.751 |
| FDC → Treg | 0.0866 | 0.0088 | -0.0777 | 0.415 | 0.761 |
| plasma → TFH | -0.0237 | -0.0144 | 0.0093 | 0.423 | 0.769 |
| LEC → CD4T | 0.4641 | 0.3743 | -0.0898 | 0.424 | 0.769 |
| B → CD4T | 9.8705 | 8.3895 | -1.4810 | 0.431 | 0.776 |
| CAF → LEC | -0.0112 | -0.0074 | 0.0037 | 0.433 | 0.777 |
| NK → mast | -0.0018 | 0.0059 | 0.0078 | 0.438 | 0.780 |
| B → granulocyte | 2.0885 | 1.4342 | -0.6543 | 0.440 | 0.780 |
| CD8T → plasma | 3.0850 | 2.5930 | -0.4920 | 0.445 | 0.784 |
| stromal → macrophages | 2.5864 | 3.0408 | 0.4543 | 0.453 | 0.789 |
| CD8T → TFH | 0.0965 | 0.1831 | 0.0866 | 0.453 | 0.789 |
| CD4T → CD8T | 11.3186 | 13.0613 | 1.7427 | 0.457 | 0.789 |
| B → plasma | 4.2174 | 3.2979 | -0.9196 | 0.460 | 0.789 |
| gdT → granulocyte | -0.0038 | -0.0055 | -0.0017 | 0.460 | 0.789 |
| mast → vasculature | 0.0731 | 0.0530 | -0.0201 | 0.468 | 0.795 |
| B → gdT | 1.2005 | 1.6284 | 0.4279 | 0.469 | 0.795 |
| LEC → gdT | 0.0503 | 0.0288 | -0.0215 | 0.474 | 0.798 |
| DC → B | 1.9816 | 1.7128 | -0.2688 | 0.479 | 0.798 |
| LEC → stromal | 0.6265 | 0.5142 | -0.1123 | 0.482 | 0.798 |
| CD4T → CD4T | 10.5040 | 12.1298 | 1.6258 | 0.485 | 0.798 |
| stromal → Treg | 2.0974 | 2.3943 | 0.2969 | 0.485 | 0.798 |
| plasma → CAF | 0.0373 | 0.0707 | 0.0333 | 0.486 | 0.798 |
| LEC → macrophages | 0.7226 | 0.5827 | -0.1398 | 0.503 | 0.819 |

Continued on next page

| Cell type pair | LN benign | LN met-adjacent | Effect size | p-value | adjusted p-value |
|---|---|---|---|---|---|
| LEC → VSMC_myofibroblast | -0.0113 | -0.0178 | -0.0065 | 0.504 | 0.819 |
| stromal → LEC | 1.6314 | 1.3807 | -0.2507 | 0.513 | 0.821 |
| vasculature → vasculature | 2.2148 | 2.4176 | 0.2029 | 0.513 | 0.821 |
| NK → TFH | -0.0109 | -0.0071 | 0.0038 | 0.514 | 0.821 |
| VSMC_myofibroblast → granulocyte | -0.0151 | -0.0112 | 0.0039 | 0.518 | 0.821 |
| plasma → granulocyte | 0.0273 | 0.0043 | -0.0230 | 0.521 | 0.821 |
| stromal → NK | 0.7250 | 0.8524 | 0.1274 | 0.528 | 0.821 |
| LEC → FDC | -0.0259 | -0.0069 | 0.0190 | 0.528 | 0.821 |
| Treg → B | 2.2187 | 2.5509 | 0.3322 | 0.531 | 0.821 |
| CAF → plasma | 0.0521 | 0.0343 | -0.0178 | 0.532 | 0.821 |
| macrophages → CD4T | 2.3184 | 2.0746 | -0.2438 | 0.536 | 0.821 |
| mast → mast | 0.0361 | 0.0500 | 0.0139 | 0.537 | 0.821 |
| NK → CD4T | 0.2592 | 0.3000 | 0.0408 | 0.538 | 0.821 |
| LEC → DC | 0.3984 | 0.3382 | -0.0602 | 0.540 | 0.821 |
| DC → plasma | 0.7166 | 0.5996 | -0.1170 | 0.542 | 0.821 |
| TFH → stromal | 0.0165 | 0.0322 | 0.0157 | 0.546 | 0.822 |
| VSMC_myofibroblast → CD8T | 0.3084 | 0.4851 | 0.1768 | 0.548 | 0.822 |
| CAF → NK | 0.0040 | -0.0040 | -0.0080 | 0.554 | 0.825 |
| macrophages → granulocyte | 1.4077 | 1.1319 | -0.2759 | 0.555 | 0.825 |
| stromal → stromal | 2.6663 | 3.0825 | 0.4162 | 0.560 | 0.828 |
| stromal → DC | 1.9368 | 2.1571 | 0.2204 | 0.563 | 0.828 |
| CD8T → NK | 1.6675 | 1.8836 | 0.2161 | 0.566 | 0.830 |
| NK → CAF | 0.0112 | 0.0015 | -0.0098 | 0.570 | 0.830 |
| stromal → VSMC_myofibroblast | 0.8325 | 1.0578 | 0.2253 | 0.572 | 0.830 |
| Treg → FDC | 0.0739 | 0.0351 | -0.0389 | 0.585 | 0.841 |
| gdT → VSMC_myofibroblast | -0.0027 | 0.0040 | 0.0067 | 0.585 | 0.841 |
| granulocyte → CAF | -0.0082 | -0.0058 | 0.0024 | 0.589 | 0.841 |
| plasma → DC | 0.4942 | 0.4186 | -0.0755 | 0.592 | 0.841 |
| VSMC_myofibroblast → B | 0.2567 | 0.3786 | 0.1219 | 0.593 | 0.841 |
| B → CD8T | 7.5371 | 6.6969 | -0.8402 | 0.596 | 0.841 |
| stromal → FDC | -0.0274 | -0.0102 | 0.0171 | 0.600 | 0.844 |
| stromal → granulocyte | 1.5802 | 1.3583 | -0.2219 | 0.613 | 0.844 |
| TFH → CD4T | 0.0442 | 0.0532 | 0.0090 | 0.614 | 0.844 |
| macrophages → TFH | -0.0092 | 0.0048 | 0.0140 | 0.617 | 0.844 |
| DC → NK | 0.5480 | 0.6276 | 0.0796 | 0.621 | 0.844 |
| VSMC_myofibroblast → stromal | 0.3996 | 0.5114 | 0.1118 | 0.624 | 0.844 |
| CD4T → stromal | 8.9838 | 10.1661 | 1.1823 | 0.625 | 0.844 |
| B → LEC | 1.3786 | 1.7037 | 0.3251 | 0.627 | 0.844 |

Continued on next page

| Cell type pair | LN benign | LN met-adjacent | Effect size | p-value | adjusted p-value |
|---|---|---|---|---|---|
| DC → Treg | 1.9519 | 1.7424 | -0.2095 | 0.630 | 0.844 |
| LEC → Treg | 0.2624 | 0.2258 | -0.0367 | 0.631 | 0.844 |
| CD8T → LEC | 0.8270 | 0.6981 | -0.1289 | 0.631 | 0.844 |
| NK → stromal | 0.2422 | 0.2805 | 0.0383 | 0.637 | 0.844 |
| B → NK | 1.6966 | 2.0093 | 0.3127 | 0.640 | 0.844 |
| FDC → plasma | -0.0133 | -0.0274 | -0.0141 | 0.645 | 0.844 |
| plasma → gdT | 0.1408 | 0.1744 | 0.0335 | 0.645 | 0.844 |
| macrophages → Treg | 1.5035 | 1.6262 | 0.1227 | 0.649 | 0.844 |
| vasculature → VSMC_myofibroblast | 0.6228 | 0.7334 | 0.1105 | 0.650 | 0.844 |
| mast → VSMC_myofibroblast | -0.0047 | -0.0040 | 0.0007 | 0.652 | 0.844 |
| FDC → VSMC_myofibroblast | -0.1270 | -0.0908 | 0.0362 | 0.655 | 0.844 |
| B → vasculature | 3.4809 | 2.9396 | -0.5413 | 0.660 | 0.844 |
| VSMC_myofibroblast → NK | 0.0048 | 0.0203 | 0.0156 | 0.663 | 0.844 |
| LEC → NK | 0.1051 | 0.1279 | 0.0228 | 0.663 | 0.844 |
| Treg → mast | 0.1238 | 0.1015 | -0.0222 | 0.668 | 0.844 |
| gdT → mast | -0.0055 | -0.0068 | -0.0013 | 0.670 | 0.844 |
| TFH → NK | -0.0196 | -0.0097 | 0.0098 | 0.670 | 0.844 |
| CD4T → CAF | 0.9633 | 0.8119 | -0.1513 | 0.674 | 0.844 |
| B → CAF | 0.4320 | 0.5517 | 0.1197 | 0.676 | 0.844 |
| VSMC_myofibroblast → plasma | 0.1781 | 0.2305 | 0.0524 | 0.677 | 0.844 |
| macrophages → stromal | 2.2978 | 2.1258 | -0.1720 | 0.678 | 0.844 |
| CAF → B | 0.0607 | 0.0521 | -0.0086 | 0.679 | 0.844 |
| NK → LEC | 0.0863 | 0.0733 | -0.0130 | 0.683 | 0.846 |
| NK → DC | 0.2092 | 0.2327 | 0.0235 | 0.690 | 0.851 |
| TFH → gdT | -0.0125 | -0.0153 | -0.0028 | 0.707 | 0.868 |
| Treg → DC | 2.3040 | 2.5314 | 0.2273 | 0.711 | 0.870 |
| DC → VSMC_myofibroblast | 0.2115 | 0.2788 | 0.0673 | 0.722 | 0.879 |
| CD4T → plasma | 5.7259 | 5.2870 | -0.4389 | 0.726 | 0.882 |
| Treg → plasma | 1.4173 | 1.2892 | -0.1281 | 0.740 | 0.885 |
| macrophages → macrophages | 2.0569 | 1.9151 | -0.1417 | 0.744 | 0.885 |
| CD8T → FDC | -0.0703 | -0.0354 | 0.0350 | 0.745 | 0.885 |
| TFH → CD8T | 0.0250 | 0.0359 | 0.0109 | 0.745 | 0.885 |
| B → macrophages | 4.6043 | 4.2060 | -0.3983 | 0.746 | 0.885 |
| mast → gdT | -0.0056 | -0.0065 | -0.0010 | 0.746 | 0.885 |
| stromal → vasculature | 2.8076 | 2.6271 | -0.1805 | 0.749 | 0.886 |
| macrophages → B | 2.0545 | 1.9370 | -0.1175 | 0.755 | 0.886 |
| DC → FDC | 0.1695 | 0.1419 | -0.0277 | 0.756 | 0.886 |
| FDC → CD8T | -0.0038 | -0.0350 | -0.0312 | 0.761 | 0.886 |
| LEC → plasma | 0.0670 | 0.0846 | 0.0176 | 0.767 | 0.886 |
| stromal → CD8T | 2.7442 | 2.8897 | 0.1456 | 0.768 | 0.886 |
| CD4T → FDC | 0.1219 | 0.0296 | -0.0924 | 0.770 | 0.886 |

Continued on next page

| Cell type pair | LN benign | LN met-adjacent | Effect size | p-value | adjusted p-value |
|---|---|---|---|---|---|
| vasculature → Treg | 0.8774 | 0.8287 | -0.0487 | 0.770 | 0.886 |
| TFH → plasma | -0.0078 | -0.0110 | -0.0031 | 0.777 | 0.886 |
| Treg → vasculature | 0.9767 | 1.0676 | 0.0909 | 0.777 | 0.886 |
| LEC → CD8T | 0.3773 | 0.3472 | -0.0301 | 0.779 | 0.886 |
| macrophages → plasma | 1.3345 | 1.4189 | 0.0844 | 0.781 | 0.886 |
| Treg → Treg | 2.1120 | 2.2804 | 0.1684 | 0.784 | 0.886 |
| TFH → B | 0.0665 | 0.0604 | -0.0061 | 0.797 | 0.898 |
| macrophages → CAF | 0.1996 | 0.1833 | -0.0164 | 0.818 | 0.918 |
| mast → NK | -0.0027 | -0.0011 | 0.0017 | 0.825 | 0.920 |
| gdT → Treg | 0.1229 | 0.1291 | 0.0063 | 0.826 | 0.920 |
| LEC → CAF | 0.0038 | -0.0017 | -0.0054 | 0.833 | 0.925 |
| NK → macrophages | 0.2573 | 0.2750 | 0.0177 | 0.850 | 0.937 |
| DC → TFH | 0.3359 | 0.3644 | 0.0285 | 0.852 | 0.937 |
| Treg → CD4T | 2.7842 | 2.9002 | 0.1159 | 0.856 | 0.937 |
| FDC → NK | -0.0805 | -0.0715 | 0.0091 | 0.856 | 0.937 |
| NK → B | 0.2384 | 0.2515 | 0.0131 | 0.858 | 0.937 |
| vasculature → NK | 0.3053 | 0.3205 | 0.0152 | 0.867 | 0.940 |
| NK → vasculature | 0.1339 | 0.1420 | 0.0081 | 0.870 | 0.940 |
| FDC → gdT | -0.0255 | -0.0280 | -0.0024 | 0.872 | 0.940 |
| VSMC_myofibroblast → gdT | -0.0048 | -0.0023 | 0.0025 | 0.874 | 0.940 |
| CD4T → vasculature | 5.1112 | 5.3009 | 0.1897 | 0.892 | 0.949 |
| B → Treg | 7.1337 | 7.3266 | 0.1929 | 0.899 | 0.949 |
| stromal → B | 2.8371 | 2.7715 | -0.0656 | 0.899 | 0.949 |
| B → stromal | 6.4043 | 6.1962 | -0.2081 | 0.901 | 0.949 |
| CD4T → macrophages | 8.5122 | 8.7248 | 0.2127 | 0.904 | 0.949 |
| LEC → LEC | 0.9461 | 0.9893 | 0.0432 | 0.904 | 0.949 |
| FDC → CD4T | 0.0402 | 0.0366 | -0.0036 | 0.907 | 0.949 |
| CD8T → VSMC_myofibroblast | 0.8260 | 0.8842 | 0.0582 | 0.908 | 0.949 |
| CAF → VSMC_myofibroblast | 0.0011 | 0.0034 | 0.0024 | 0.910 | 0.949 |
| Treg → LEC | 0.1989 | 0.2153 | 0.0163 | 0.912 | 0.949 |
| macrophages → NK | 0.8030 | 0.7744 | -0.0286 | 0.918 | 0.951 |
| macrophages → CD8T | 2.0191 | 1.9860 | -0.0332 | 0.927 | 0.958 |
| CD4T → LEC | 1.5646 | 1.5166 | -0.0480 | 0.932 | 0.958 |
| granulocyte → LEC | 0.7370 | 0.7115 | -0.0255 | 0.935 | 0.958 |
| Treg → macrophages | 1.8698 | 1.9077 | 0.0378 | 0.936 | 0.958 |
| LEC → B | 0.4061 | 0.4126 | 0.0065 | 0.955 | 0.974 |
| NK → FDC | -0.0224 | -0.0220 | 0.0004 | 0.959 | 0.975 |
| stromal → plasma | 1.5150 | 1.5332 | 0.0183 | 0.962 | 0.975 |
| CAF → granulocyte | 0.0094 | 0.0089 | -0.0005 | 0.973 | 0.983 |
| VSMC_myofibroblast → mast | -0.0048 | -0.0049 | -0.0001 | 0.980 | 0.983 |
| Treg → stromal | 2.2701 | 2.2861 | 0.0160 | 0.980 | 0.983 |

Continued on next page

| Cell type pair | LN benign | LN met-adjacent | Effect size | p-value | adjusted p-value |
|---|---|---|---|---|---|
| stromal → CD4T | 3.0179 | 3.0152 | -0.0026 | 0.996 | 0.996 |

## S9.3 HNSCC spicyR benchmark

Table S7: HNSCC TMA spicyR results (benchmark context).

| Cell type pair | Effect size | p-value | adjusted p-value |
|---|---|---|---|
| vasculature → LEC | -128.8321 | 0.070 | 0.999 |
| NK → VSMC_myofibroblast | 170.7245 | 0.070 | 0.999 |
| LEC → vasculature | -129.0625 | 0.071 | 0.999 |
| mast → mast | 855.6196 | 0.072 | 0.999 |
| VSMC_myofibroblast → NK | 169.0685 | 0.073 | 0.999 |
| mast → granulocyte | -337.3361 | 0.080 | 0.999 |
| granulocyte → mast | -334.4272 | 0.083 | 0.999 |
| stromal → B | 50.7820 | 0.094 | 0.999 |
| B → stromal | 50.1138 | 0.098 | 0.999 |
| granulocyte → TFH | 174.4088 | 0.127 | 0.999 |
| TFH → granulocyte | 175.9773 | 0.130 | 0.999 |
| FDC → Treg | -118.4221 | 0.140 | 0.999 |
| Treg → FDC | -113.9966 | 0.153 | 0.999 |
| mast → CD8T | -82.0483 | 0.154 | 0.999 |
| LEC → stromal | -69.0542 | 0.157 | 0.999 |
| NK → NK | 243.6599 | 0.158 | 0.999 |
| FDC → VSMC_myofibroblast | 140.0783 | 0.159 | 0.999 |
| VSMC_myofibroblast → FDC | 139.9504 | 0.159 | 0.999 |
| TFH → LEC | 146.6103 | 0.161 | 0.999 |
| stromal → LEC | -67.9163 | 0.164 | 0.999 |
| vasculature → CAF | -188.4217 | 0.170 | 0.999 |
| LEC → TFH | 142.2029 | 0.174 | 0.999 |
| VSMC_myofibroblast → Treg | 94.1867 | 0.177 | 0.999 |
| Treg → VSMC_myofibroblast | 94.8254 | 0.178 | 0.999 |
| CD8T → mast | -75.4413 | 0.182 | 0.999 |
| DC → FDC | 97.2959 | 0.185 | 0.999 |
| FDC → DC | 100.1006 | 0.187 | 0.999 |
| CAF → vasculature | -179.0372 | 0.196 | 0.999 |
| CD4T → TFH | 75.7628 | 0.211 | 0.999 |
| plasma → CD4T | -53.6925 | 0.221 | 0.999 |
| TFH → CD4T | 74.0555 | 0.222 | 0.999 |
| CD4T → plasma | -52.7265 | 0.229 | 0.999 |
| macrophages → B | 44.7878 | 0.236 | 0.999 |
| B → macrophages | 44.8161 | 0.237 | 0.999 |
| granulocyte → CD8T | -47.6498 | 0.256 | 0.999 |

Continued on next page

| Cell type pair | Effect size | p-value | adjusted p-value |
|---|---|---|---|
| NK → FDC | 123.6373 | 0.266 | 0.999 |
| FDC → NK | 120.4809 | 0.269 | 0.999 |
| mast → DC | -67.9247 | 0.271 | 0.999 |
| CD8T → granulocyte | -45.5289 | 0.281 | 0.999 |
| TFH → TFH | -381.9967 | 0.297 | 0.999 |
| CD4T → vasculature | -26.1400 | 0.298 | 0.999 |
| stromal → DC | 29.2097 | 0.301 | 0.999 |
| DC → plasma | -53.2747 | 0.304 | 0.999 |
| FDC → plasma | -97.7280 | 0.310 | 0.999 |
| plasma → DC | -52.4200 | 0.310 | 0.999 |
| DC → stromal | 27.9670 | 0.316 | 0.999 |
| CD4T → granulocyte | -39.3134 | 0.322 | 0.999 |
| granulocyte → CD4T | -39.0374 | 0.327 | 0.999 |
| stromal → mast | -52.9226 | 0.329 | 0.999 |
| vasculature → CD4T | -24.7693 | 0.329 | 0.999 |
| granulocyte → DC | -67.4760 | 0.332 | 0.999 |
| plasma → FDC | -92.9093 | 0.333 | 0.999 |
| DC → granulocyte | -66.8269 | 0.338 | 0.999 |
| Treg → granulocyte | -48.8358 | 0.342 | 0.999 |
| vasculature → TFH | 73.4613 | 0.352 | 0.999 |
| granulocyte → Treg | -47.3908 | 0.358 | 0.999 |
| vasculature → DC | -46.4014 | 0.362 | 0.999 |
| TFH → vasculature | 70.2531 | 0.371 | 0.999 |
| DC → vasculature | -45.0727 | 0.379 | 0.999 |
| mast → stromal | -47.4656 | 0.380 | 0.999 |
| granulocyte → vasculature | -132.3022 | 0.381 | 0.999 |
| vasculature → granulocyte | -131.0432 | 0.385 | 0.999 |
| DC → mast | -55.8717 | 0.392 | 0.999 |
| plasma → stromal | 28.8018 | 0.393 | 0.999 |
| VSMC_myofibroblast → mast | -105.3777 | 0.394 | 0.999 |
| gdT → Treg | -99.5772 | 0.394 | 0.999 |
| VSMC_myofibroblast → CD4T | -50.1407 | 0.396 | 0.999 |
| Treg → gdT | -98.6569 | 0.397 | 0.999 |
| mast → macrophages | 66.6682 | 0.402 | 0.999 |
| Treg → plasma | -33.9866 | 0.403 | 0.999 |
| CAF → B | -46.4975 | 0.406 | 0.999 |
| granulocyte → macrophages | 63.2631 | 0.406 | 0.999 |
| macrophages → mast | 65.3950 | 0.412 | 0.999 |
| B → CAF | -45.9066 | 0.413 | 0.999 |

Continued on next page

| Cell type pair | Effect size | p-value | adjusted p-value |
|---|---|---|---|
| DC → NK | -54.1061 | 0.414 | 0.999 |
| stromal → VSMC_myofibroblast | -51.6869 | 0.415 | 0.999 |
| NK → DC | -54.4676 | 0.415 | 0.999 |
| VSMC_myofibroblast → CD8T | -44.3164 | 0.417 | 0.999 |
| B → DC | 55.2456 | 0.421 | 0.999 |
| DC → B | 56.5711 | 0.421 | 0.999 |
| CD8T → vasculature | -24.4957 | 0.421 | 0.999 |
| VSMC_myofibroblast → stromal | -50.4435 | 0.426 | 0.999 |
| mast → VSMC_myofibroblast | -98.9768 | 0.427 | 0.999 |
| macrophages → Treg | 24.6216 | 0.427 | 0.999 |
| TFH → B | -68.6455 | 0.427 | 0.999 |
| plasma → Treg | -32.1760 | 0.428 | 0.999 |
| B → TFH | -66.7206 | 0.437 | 0.999 |
| macrophages → FDC | 57.1178 | 0.438 | 0.999 |
| macrophages → granulocyte | 60.0995 | 0.439 | 0.999 |
| vasculature → CD8T | -23.9588 | 0.439 | 0.999 |
| granulocyte → NK | -82.4936 | 0.440 | 0.999 |
| FDC → macrophages | 57.0158 | 0.441 | 0.999 |
| Treg → macrophages | 23.5658 | 0.441 | 0.999 |
| vasculature → mast | -65.2368 | 0.441 | 0.999 |
| granulocyte → granulocyte | 313.4256 | 0.445 | 0.999 |
| stromal → plasma | 25.7593 | 0.447 | 0.999 |
| mast → vasculature | -66.1565 | 0.447 | 0.999 |
| gdT → gdT | 305.8428 | 0.452 | 0.999 |
| DC → TFH | 50.6166 | 0.453 | 0.999 |
| DC → DC | 91.1231 | 0.454 | 0.999 |
| CD4T → FDC | 44.4585 | 0.461 | 0.999 |
| VSMC_myofibroblast → plasma | 79.1890 | 0.462 | 0.999 |
| NK → granulocyte | -78.8083 | 0.462 | 0.999 |
| CD4T → VSMC_myofibroblast | -43.9796 | 0.469 | 0.999 |
| TFH → DC | 48.4848 | 0.471 | 0.999 |
| stromal → vasculature | -21.4491 | 0.478 | 0.999 |
| LEC → granulocyte | -97.5484 | 0.479 | 0.999 |
| CD8T → VSMC_myofibroblast | -39.3360 | 0.479 | 0.999 |
| CAF → mast | -115.1047 | 0.490 | 0.999 |

Continued on next page

| Cell type pair | Effect size | p-value | adjusted p-value |
|---|---|---|---|
| mast → CAF | -115.7324 | 0.492 | 0.999 |
| plasma → VSMC_myofibroblast | 76.2103 | 0.492 | 0.999 |
| stromal → gdT | 36.4844 | 0.492 | 0.999 |
| vasculature → stromal | -21.0667 | 0.495 | 0.999 |
| TFH → VSMC_myofibroblast | 70.8650 | 0.495 | 0.999 |
| gdT → stromal | 35.7070 | 0.501 | 0.999 |
| DC → CD4T | 38.5328 | 0.503 | 0.999 |
| NK → Treg | 50.0171 | 0.504 | 0.999 |
| CD4T → DC | 37.2320 | 0.510 | 0.999 |
| Treg → NK | 48.6089 | 0.515 | 0.999 |
| FDC → CD4T | 38.8817 | 0.519 | 0.999 |
| granulocyte → LEC | -88.6479 | 0.520 | 0.999 |
| gdT → plasma | -58.8904 | 0.525 | 0.999 |
| B → B | 57.8839 | 0.525 | 0.999 |
| macrophages → vasculature | -20.8569 | 0.527 | 0.999 |
| mast → CD4T | -39.8565 | 0.529 | 0.999 |
| plasma → gdT | -57.7289 | 0.533 | 0.999 |
| macrophages → stromal | 11.3071 | 0.534 | 0.999 |
| stromal → macrophages | 11.4974 | 0.544 | 0.999 |
| VSMC_myofibroblast → TFH | 62.6628 | 0.547 | 0.999 |
| LEC → plasma | 49.5883 | 0.548 | 0.999 |
| vasculature → FDC | 47.5151 | 0.549 | 0.999 |
| CAF → NK | -71.9212 | 0.552 | 0.999 |
| VSMC_myofibroblast → vasculature | -87.6623 | 0.554 | 0.999 |
| granulocyte → CAF | -78.1180 | 0.555 | 0.999 |
| LEC → DC | -34.1302 | 0.555 | 0.999 |
| DC → CD8T | -22.9607 | 0.555 | 0.999 |
| CAF → granulocyte | -77.6677 | 0.556 | 0.999 |
| FDC → vasculature | 46.0829 | 0.562 | 0.999 |
| vasculature → macrophages | -18.9819 | 0.563 | 0.999 |
| CD4T → mast | -38.7995 | 0.564 | 0.999 |
| CD8T → DC | -22.3743 | 0.569 | 0.999 |
| gdT → VSMC_myofibroblast | 54.4061 | 0.584 | 0.999 |
| VSMC_myofibroblast → gdT | 53.9432 | 0.587 | 0.999 |
| vasculature → VSMC_myofibroblast | -81.5061 | 0.594 | 0.999 |
| LEC → CD8T | -24.3527 | 0.598 | 0.999 |
| CD8T → LEC | -23.9204 | 0.601 | 0.999 |
| B → CD8T | 29.3687 | 0.602 | 0.999 |
| CD8T → B | 28.7713 | 0.608 | 0.999 |

Continued on next page

| Cell type pair | Effect size | p-value | adjusted p-value |
|---|---|---|---|
| mast → gdT | 75.7785 | 0.614 | 0.999 |
| B → mast | -33.0905 | 0.622 | 0.999 |
| stromal → granulocyte | -24.3416 | 0.622 | 0.999 |
| gdT → LEC | 83.1668 | 0.628 | 0.999 |
| DC → LEC | -29.0373 | 0.629 | 0.999 |
| VSMC_myofibroblast → LEC | -43.0659 | 0.641 | 0.999 |
| VSMC_myofibroblast → VSMC_myofibroblast | -227.5744 | 0.641 | 0.999 |
| gdT → mast | 69.8243 | 0.644 | 0.999 |
| CAF → macrophages | 23.6610 | 0.646 | 0.999 |
| DC → CAF | -33.6411 | 0.648 | 0.999 |
| plasma → LEC | 37.7481 | 0.648 | 0.999 |
| gdT → NK | 47.6496 | 0.649 | 0.999 |
| LEC → gdT | 77.5901 | 0.651 | 0.999 |
| NK → gdT | 45.9853 | 0.660 | 0.999 |
| LEC → CAF | -60.8395 | 0.662 | 0.999 |
| CAF → DC | -32.1152 | 0.662 | 0.999 |
| granulocyte → stromal | -21.6862 | 0.664 | 0.999 |
| NK → B | 44.0675 | 0.665 | 0.999 |
| CAF → LEC | -59.6736 | 0.667 | 0.999 |
| mast → B | -28.8112 | 0.670 | 0.999 |
| CAF → plasma | 47.0034 | 0.670 | 0.999 |
| CD4T → CAF | 19.3864 | 0.672 | 0.999 |
| macrophages → CAF | 21.8960 | 0.672 | 0.999 |
| NK → LEC | -38.4815 | 0.674 | 0.999 |
| CAF → CD4T | 19.1216 | 0.675 | 0.999 |
| B → granulocyte | 26.6799 | 0.675 | 0.999 |
| LEC → mast | -45.8904 | 0.677 | 0.999 |
| B → CD4T | 30.1202 | 0.678 | 0.999 |
| LEC → VSMC_myofibroblast | -38.2114 | 0.679 | 0.999 |
| FDC → TFH | -144.9371 | 0.682 | 0.999 |
| B → NK | 41.2332 | 0.684 | 0.999 |
| FDC → LEC | 81.5173 | 0.684 | 0.999 |
| CD4T → B | 29.1310 | 0.684 | 0.999 |
| NK → CAF | -46.5595 | 0.686 | 0.999 |
| granulocyte → FDC | 43.1844 | 0.686 | 0.999 |
| vasculature → gdT | 31.0108 | 0.687 | 0.999 |
| LEC → FDC | 80.6286 | 0.688 | 0.999 |
| plasma → CAF | 43.7727 | 0.689 | 0.999 |
| macrophages → gdT | 25.5335 | 0.690 | 0.999 |
| CD8T → NK | 41.8602 | 0.692 | 0.999 |
| macrophages → TFH | 28.7107 | 0.693 | 0.999 |
| FDC → granulocyte | 41.8842 | 0.693 | 0.999 |

Continued on next page

| Cell type pair | Effect size | p-value | adjusted p-value |
|---|---|---|---|
| macrophages → CD4T | 12.3934 | 0.694 | 0.999 |
| gdT → B | -20.6575 | 0.695 | 0.999 |
| gdT → granulocyte | 34.7341 | 0.696 | 0.999 |
| granulocyte → gdT | 34.5618 | 0.697 | 0.999 |
| TFH → gdT | 48.4626 | 0.697 | 0.999 |
| TFH → FDC | -137.6888 | 0.700 | 0.999 |
| granulocyte → B | 24.1907 | 0.705 | 0.999 |
| gdT → TFH | 46.7562 | 0.708 | 0.999 |
| NK → TFH | 40.8753 | 0.708 | 0.999 |
| B → gdT | -19.7662 | 0.708 | 0.999 |
| DC → VSMC_myofibroblast | -26.2117 | 0.712 | 0.999 |
| LEC → NK | -33.5470 | 0.717 | 0.999 |
| CD4T → gdT | -21.2035 | 0.717 | 0.999 |
| stromal → CD8T | -8.0951 | 0.718 | 0.999 |
| CD4T → macrophages | 11.3810 | 0.719 | 0.999 |
| CD4T → stromal | 8.5922 | 0.719 | 0.999 |
| mast → LEC | -39.7905 | 0.720 | 0.999 |
| stromal → Treg | 8.1827 | 0.734 | 0.999 |
| stromal → CD4T | 8.1859 | 0.735 | 0.999 |
| CD8T → gdT | 24.9224 | 0.741 | 0.999 |
| gdT → vasculature | 25.3657 | 0.741 | 0.999 |
| TFH → NK | 35.6844 | 0.742 | 0.999 |
| gdT → CD4T | -19.2044 | 0.743 | 0.999 |
| CD4T → LEC | -13.8504 | 0.745 | 0.999 |
| VSMC_myofibroblast → DC | -22.9297 | 0.747 | 0.999 |
| gdT → CAF | 48.7062 | 0.747 | 0.999 |
| CAF → gdT | 48.1772 | 0.749 | 0.999 |
| NK → stromal | -14.9209 | 0.750 | 0.999 |
| gdT → CD8T | 23.9017 | 0.751 | 0.999 |
| NK → CD8T | 33.1365 | 0.754 | 0.999 |
| LEC → CD4T | -15.1146 | 0.756 | 0.999 |
| macrophages → macrophages | 14.8810 | 0.756 | 0.999 |
| plasma → vasculature | 28.9168 | 0.760 | 0.999 |
| vasculature → plasma | 28.4318 | 0.760 | 0.999 |
| gdT → macrophages | 19.3219 | 0.763 | 0.999 |
| CAF → VSMC_myofibroblast | 69.9550 | 0.765 | 0.999 |
| VSMC_myofibroblast → CAF | 69.2193 | 0.767 | 0.999 |
| CD8T → CAF | -12.9345 | 0.769 | 0.999 |
| TFH → mast | -28.4888 | 0.772 | 0.999 |
| mast → Treg | 17.4878 | 0.775 | 0.999 |
| NK → vasculature | -15.6699 | 0.775 | 0.999 |
| FDC → mast | -27.7509 | 0.781 | 0.999 |

Continued on next page

| Cell type pair | Effect size | p-value | adjusted p-value |
|---|---|---|---|
| mast → TFH | -26.8570 | 0.784 | 0.999 |
| LEC → macrophages | -18.7912 | 0.784 | 0.999 |
| vasculature → NK | -14.9825 | 0.786 | 0.999 |
| CD8T → TFH | 19.0252 | 0.787 | 0.999 |
| CAF → CD8T | -11.7430 | 0.790 | 0.999 |
| stromal → stromal | -7.8833 | 0.793 | 0.999 |
| FDC → CD8T | -15.1329 | 0.800 | 0.999 |
| Treg → LEC | -14.7647 | 0.800 | 0.999 |
| macrophages → LEC | -16.8894 | 0.803 | 0.999 |
| CD8T → stromal | -5.4441 | 0.807 | 0.999 |
| CAF → FDC | -27.5498 | 0.815 | 0.999 |
| Treg → stromal | 5.4183 | 0.815 | 0.999 |
| LEC → Treg | -13.5770 | 0.816 | 0.999 |
| macrophages → DC | -8.2284 | 0.816 | 0.999 |
| plasma → macrophages | -12.7333 | 0.818 | 0.999 |
| TFH → Treg | -15.7295 | 0.819 | 0.999 |
| Treg → TFH | -15.5606 | 0.820 | 0.999 |
| CD8T → macrophages | 7.4116 | 0.820 | 0.999 |
| TFH → CD8T | 15.8841 | 0.821 | 0.999 |
| FDC → CAF | -26.6348 | 0.821 | 0.999 |
| TFH → macrophages | 16.4771 | 0.823 | 0.999 |
| B → Treg | 16.1840 | 0.824 | 0.999 |
| CD8T → CD8T | 12.8362 | 0.825 | 0.999 |
| B → VSMC_myofibroblast | 12.1224 | 0.831 | 0.999 |
| CD4T → CD4T | 15.6060 | 0.834 | 0.999 |
| CD8T → CD4T | 13.4390 | 0.835 | 0.999 |
| macrophages → CD8T | 6.7145 | 0.835 | 0.999 |
| vasculature → B | -7.8920 | 0.839 | 0.999 |
| B → vasculature | -7.6993 | 0.840 | 0.999 |
| stromal → NK | -9.4740 | 0.841 | 0.999 |
| plasma → plasma | -28.7364 | 0.841 | 0.999 |
| Treg → B | 13.8852 | 0.847 | 0.999 |
| CD4T → CD8T | 12.3039 | 0.847 | 0.999 |
| plasma → CD8T | -8.8128 | 0.849 | 0.999 |
| LEC → LEC | 40.9005 | 0.850 | 0.999 |
| vasculature → vasculature | -22.4713 | 0.855 | 0.999 |
| VSMC_myofibroblast → B | 10.1783 | 0.857 | 0.999 |
| DC → macrophages | -6.0704 | 0.867 | 0.999 |
| NK → CD4T | 13.3152 | 0.869 | 0.999 |
| DC → gdT | -12.7295 | 0.875 | 0.999 |
| gdT → DC | -12.5286 | 0.877 | 0.999 |
| CAF → stromal | 10.6410 | 0.878 | 0.999 |

Continued on next page

| Cell type pair | Effect size | p-value | adjusted p-value |
|---|---|---|---|
| stromal → TFH | 8.4954 | 0.880 | 0.999 |
| CD4T → NK | 12.0524 | 0.881 | 0.999 |
| FDC → B | -13.2489 | 0.885 | 0.999 |
| CD8T → FDC | -8.5725 | 0.886 | 0.999 |
| VSMC_myofibroblast → macrophages | 10.2453 | 0.887 | 0.999 |
| VSMC_myofibroblast → granulocyte | 16.9758 | 0.889 | 0.999 |
| macrophages → NK | 7.7766 | 0.890 | 0.999 |
| mast → FDC | -13.5278 | 0.891 | 0.999 |
| CD8T → plasma | -6.2351 | 0.892 | 0.999 |
| NK → macrophages | 7.5562 | 0.893 | 0.999 |
| B → FDC | -12.1192 | 0.893 | 0.999 |
| macrophages → VSMC_myofibroblast | 9.6974 | 0.897 | 0.999 |
| B → plasma | 10.1475 | 0.903 | 0.999 |
| NK → plasma | -8.1674 | 0.906 | 0.999 |
| plasma → B | 9.8509 | 0.906 | 0.999 |
| stromal → CAF | 8.1459 | 0.907 | 0.999 |
| TFH → stromal | 6.6513 | 0.907 | 0.999 |
| vasculature → Treg | 3.9967 | 0.908 | 0.999 |
| granulocyte → VSMC_myofibroblast | 14.0643 | 0.909 | 0.999 |
| LEC → B | -6.8785 | 0.911 | 0.999 |
| Treg → Treg | -7.9585 | 0.915 | 0.999 |
| NK → mast | 12.4154 | 0.916 | 0.999 |
| plasma → NK | -7.2072 | 0.916 | 0.999 |
| plasma → mast | 8.2526 | 0.927 | 0.999 |
| mast → NK | 10.2423 | 0.930 | 0.999 |
| DC → Treg | 4.8876 | 0.933 | 0.999 |
| CAF → TFH | 10.5095 | 0.934 | 0.999 |
| TFH → CAF | 9.8964 | 0.938 | 0.999 |
| Treg → CD8T | -4.3065 | 0.942 | 0.999 |
| Treg → DC | 4.0842 | 0.944 | 0.999 |
| CAF → CAF | -16.4188 | 0.948 | 0.999 |
| B → LEC | -3.7053 | 0.952 | 0.999 |
| Treg → mast | 3.5859 | 0.953 | 0.999 |
| mast → plasma | 4.9601 | 0.956 | 0.999 |
| CD4T → Treg | 2.9813 | 0.960 | 0.999 |
| granulocyte → plasma | -4.0600 | 0.961 | 0.999 |
| Treg → vasculature | 1.2928 | 0.968 | 0.999 |
| CD8T → Treg | -2.2597 | 0.969 | 0.999 |
| gdT → FDC | -3.4178 | 0.973 | 0.999 |
| TFH → plasma | -2.7164 | 0.975 | 0.999 |
| FDC → gdT | -2.8637 | 0.977 | 0.999 |
| plasma → TFH | 2.4540 | 0.977 | 0.999 |
| macrophages → plasma | 1.2579 | 0.978 | 0.999 |
| FDC → FDC | 10.0892 | 0.980 | 0.999 |
| stromal → FDC | -0.9935 | 0.990 | 0.999 |

Continued on next page

| Cell type pair | Effect size | p-value | adjusted p-value |
|---|---|---|---|
| Treg → CD4T | 0.7661 | 0.990 | 0.999 |
| CAF → Treg | 0.8455 | 0.990 | 0.999 |
| FDC → stromal | 0.6394 | 0.993 | 0.999 |
| plasma → granulocyte | -0.3898 | 0.996 | 0.999 |
| Treg → CAF | 0.0605 | 0.999 | 0.999 |

## S9.4 HNSCC $p$-value distribution comparison

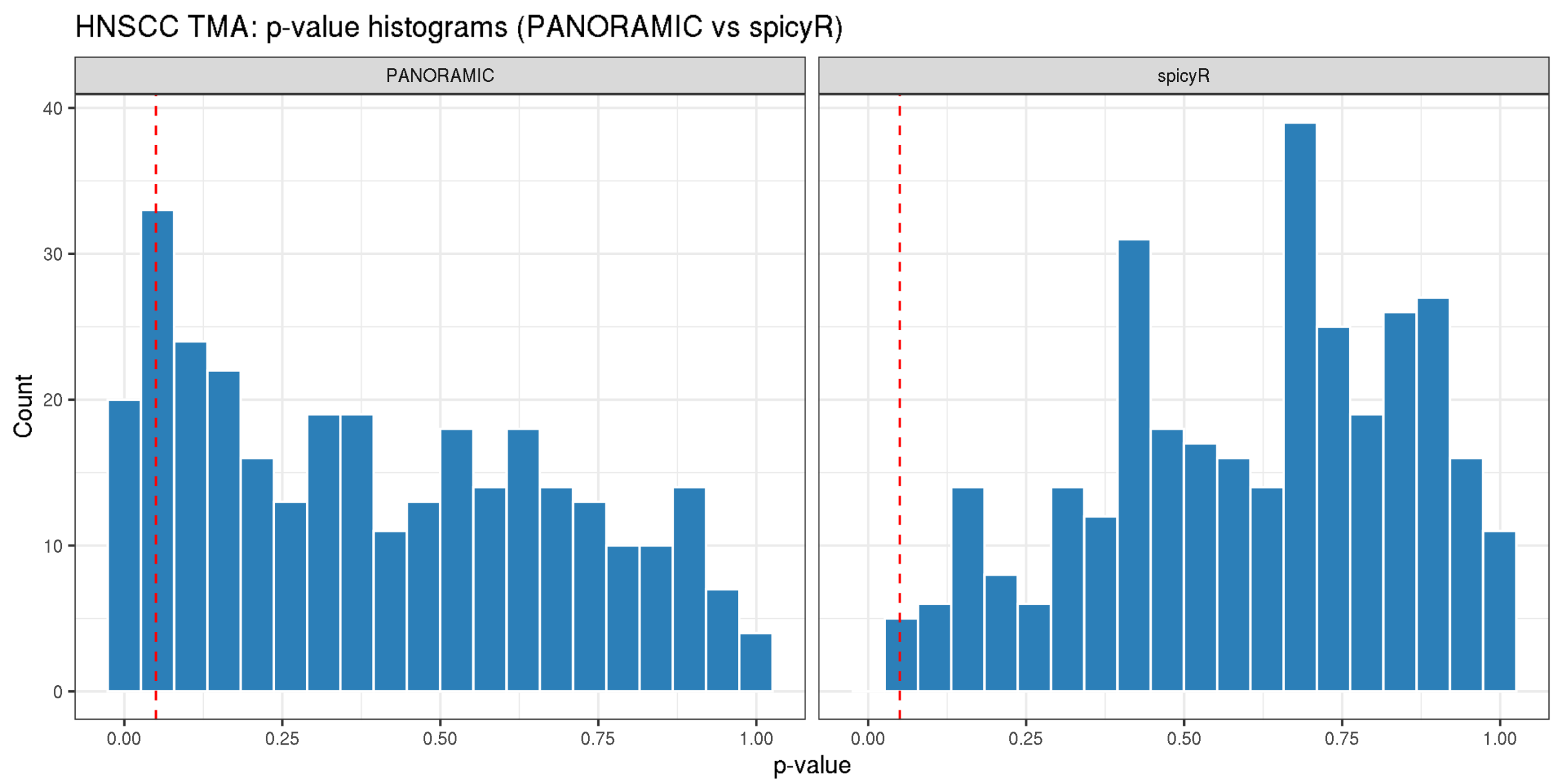


Figure S9: P-value distributions for the HNSCC TMA differential colocalization analysis comparing PANORAMIC and spicyR. Histograms show nominal p-values across tested directional cell-type pairs for the benign lymph node versus tumor-adjacent lymph node comparison. The red dashed vertical line marks $p = 0.05$. PANORAMIC shows enrichment of small p-values with a broad background across the remaining range, whereas spicyR produces fewer small p-values and a concentration of larger p-values.

# References

A. Baddeley, E. Rubak, and R. Turner. *Spatial Point Patterns: Methodology and Applications with R.* Chapman and Hall/CRC Press, Boca Raton, FL, 2015. ISBN 978-1482210200.

N. P. Canete, S. S. Iyengar, J. T. Ormerod, H. Baharlou, A. N. Harman, and E. Patrick. spicyr: spatial analysis ofin situcytometry data in r. *Bioinformatics*, 38(11):3099–3105, May 2022. ISSN 1367-4803, 1367-4811. doi: 10.1093/bioinformatics/btac268.

R. Dries, Q. Zhu, R. Dong, C.-H. L. Eng, H. Li, K. Liu, Y. Fu, T. Zhao, A. Sarkar, F. Bao, R. George, N. Pierson, L. Cai, and G.-C. Yuan. Giotto: a toolbox for integrative analysis and visualization of spatial expression data. *Genome Biology*, 22(1):78, 2021. doi: 10.1186/s13059-021-02286-2.

M. Haist, M.-A. Baertsch, N. E. Reticker-Flynn, G. Lu, T. N. Kempchen, P. Chu, G. Vazquez, H. Chen, J. B. Sunwoo, W. Zhang, E. Laseinde, B. Adami, S. Zimmer, J. Kaufman, Q. T. Le, A. J. Gentles, C. S. Kong, S. K. Plevritis, Y. Goltsev, J. W. Hickey, and G. P. Nolan. Lymph node colonization induces tissue remodeling via immunosuppressive fibroblast-myeloid cell niches supporting metastatic tolerance. *Cancer Cell*, 44(3):604–623.e9, 2026. doi: 10.1016/j.ccell.2026.01.003.

J. M. Loh. A valid and fast spatial bootstrap for correlation functions. *The Astrophysical Journal*, 681(1): 726–734, July 2008. ISSN 0004-637X, 1538-4357. doi: 10.1086/588631.

J. M. Loh. Bootstrapping an inhomogeneous point process. *Journal of Statistical Planning and Inference*, 140(3):734–749, Mar. 2010. ISSN 03783758. doi: 10.1016/j.jspi.2009.09.003.

G. Palla, D. S. Fischer, A. Regev, and F. J. Theis. Spatial components of molecular tissue biology. *Nature Biotechnology*, 40(3):308–318, Mar. 2022. ISSN 1087-0156, 1546-1696. doi: 10.1038/s41587-021-01182-1.

B. D. Ripley. The second-order analysis of stationary point processes. *Journal of Applied Probability*, June 1976.

C. M. Schürch, S. S. Bhate, G. L. Barlow, D. J. Phillips, L. Noti, I. Zlobec, P. Chu, S. Black, J. Demeter, D. R. McIlwain, S. C. Kimmey, A. C. Cote, C. Mengus, A. E. Moor, W. J. Greenleaf, and G. P. Nolan. Coordinated cellular neighborhoods orchestrate antitumoral immunity at the colorectal cancer invasive front. *Cell*, 182(5):1341–1359.e19, 2020. doi: 10.1016/j.cell.2020.07.005.

V. A. Traag, L. Waltman, and N. J. van Eck. From louvain to leiden: guaranteeing well-connected communities. *Scientific Reports*, 9(1):5233, 2019. doi: 10.1038/s41598-019-41695-z.
